# Drp1 acetylation mediated by CDK5-AMPK-GCN5L1 axis promotes cerebral ischemic injury via facilitating mitochondrial fission

**DOI:** 10.1186/s10020-024-00948-y

**Published:** 2024-10-10

**Authors:** Jiejie Zhang, Shan Wang, Haitao Zhang, Xiaotong Yang, Xin Ren, Lei Wang, Yihan Yang, Yi Yang, Ya Wen

**Affiliations:** 1 Department of Neurology, The Second Hospital of Hebei Medical University, 215 Hepingxi Road, Shijiazhuang, 050000 Hebei China; 2 Neurological Laboratory of Hebei Province, Shijiazhuang, Hebei China; 3 Department of Human Anatomy, Institute of Medicine and Health, Hebei Medical University, Shijiazhuang, 050017 Hebei China

**Keywords:** CDK5, AMPK, Drp1, GCN5L1, Acetylation, Mitochondrial fission, Neuronal cells, Ischemic stroke

## Abstract

The aberrant acetylation of mitochondrial proteins is involved in the pathogenesis of multiple diseases including neurodegenerative diseases and cerebral ischemic injury. Previous studies have shown that depletion of mitochondrial NAD+, which is necessary for mitochondrial deacetylase activity, leads to decreased activity of mitochondrial deacetylase and thus causes hyperacetylation of mitochondrial proteins in ischemic brain tissues, which results in altered mitochondrial dynamics. However, it remains largely unknown about how mitochondrial dynamics-related protein Drp1 is acetylated in ischemic neuronal cells and brain tissues. Here, we showed that Drp1 and GCN5L1 expression was up-regulated in OGD-treated neuronal cells and ischemic brain tissues induced by dMCAO, accompanied by the increased mitochondrial fission, mtROS accumulation, and cell apoptosis. Further, we confirmed that ischemia/hypoxia promoted Drp1 interaction with GCN5L1 in neuronal cells and brain tissues. GCN5L1 knockdown attenuated, while its overexpression enhanced Drp1 acetylation and mitochondrial fission, indicating that GCN5L1 plays a crucial role in ischemia/hypoxia-induced mitochondrial fission by acetylating Drp1. Mechanistically, ischemia/hypoxia induced Drp1 phosphorylation by CDK5 upregulation-mediated activation of AMPK in neuronal cells, which in turn facilitated the interaction of GCN5L1 with Drp1, thus enhancing Drp1 acetylation and mitochondrial fission. Accordingly, inhibition of AMPK alleviated ischemia/hypoxia- induced Drp1 acetylation and mitochondrial fission and protected brain tissues from ischemic damage. These findings provide a novel insight into the functional roles of GCN5L1 in regulating Drp1 acetylation and identify a previously unrecognized CDK5-AMPK-GCN5L1 pathway that mediates the acetylation of Drp1 in ischemic brain tissues.

**Supplementary Information:**

The online version contains supplementary material available at 10.1186/s10020-024-00948-y.

## Introduction

Lack of blood and oxygen supply to the brain caused by brain blood circulation disorders triggers complex pathophysiological processes, such as oxidative stress, inflammation, calcium overload, and neuronal cell death, which are the main mechanisms responsible for cerebral ischemia/hypoxia injury(He et al. 2020; Khoshnam et al. 2017). As the most energy-demanding organ in the human body, the brain, especially neuron, is highly vulnerable to ischemia/hypoxia and quickly develops dysfunction after ischemic stroke(He et al. 2020). Mitochondria are one of the most sensitive organelles to ischemic conditions, and are thus certainly involved in the cerebral ischemia/hypoxia injury(Klimova, Long, Scafidi, et al. 2019). Following ischemic stroke, ischemia/hypoxia-induced mitochondrial dysfunction leads to the ATP depletion and overproduction of reactive oxidative species (ROS), which triggers cascade response of brain to ischemia/hypoxia injury(He et al. 2020). Therefore, elucidation of novel molecular pathways that result in structural and functional alteration of mitochondria in the response to ischemia/hypoxia is essential for the prevention and treatment of ischemic stroke.

Mitochondrial dysfunction is tightly related to mitochondrial morphology, which is dynamically modulated by the balance of mitochondrial fusion and fission, namely mitochondrial dynamics(Pokharel et al. 2024). Imbalanced mitochondrial dynamics directly contributes to the pathogenesis of mitochondrial dysfunction(Li et al. 2023). Accumulating evidence has indicated that mitochondrial dynamics are regulated by the relative activities of mitochondrial fission and fusion proteins. The dynamin-like GTPases mitofusin 1 and 2 (Mfn1, Mfn2) regulate the mitochondrial fusion, whereas dynamin-related protein 1 (Drp1) mediates the regulation of mitochondrial fission. A recent report showed that activation of Drp1 and mitochondrial fission leads to ischemic injury in the brain(Flippo et al. 2018), indicating that Drp1-mediated mitochondrial fission plays an important role in cerebral ischemia/hypoxia injury. It is well known that Drp1 activity is regulated by several post-translational modifications including phosphorylation, acetylation and S-nitrosylation(Adaniya et al. 2019; Hu et al. 2020). Moreover, post-translational modifications of Drp1 are linked to mitochondrial dysfunction-mediated neuronal cell death(Dowding et al. 2014). More importantly, phosphorylation of Drp1 at Ser616 by CDK1/cyclin B, ERK1/2, PKC, and CaMKII promotes mitochondrial fission(Adaniya et al. 2019; Breitzig et al. 2018). Drp1 acetylation is a new post-translational modification of Drp1 in cardiomyocytes, and this modification is required for increased Drp1 phosphorylation at Ser616, which leads to increased activity of Drp1(Hu et al. 2020). Despite considerable progress elucidating the molecular regulation of Drp1 activity, little was known about whether and how Drp1 is acetylated in ischemic/hypoxic neuronal cells and brain tissues. Moreover, the upstream signaling regulating this process remains to be further explored.

General control of amino acid synthesis 5 (GCN5) like-1 (GCN5L1) is a novel gene that shares sequence homology with the histone acetyltransferase Gcn5(Scott et al. 2018; Wu et al. 2021). GCN5L1 is mitochondria-enriched and regulates mitochondrial protein acetylation, cellular bioenergetics, ROS generation, and organelle positioning in a number of diverse cell types(Manning et al. 2019; Scott et al. 2012; Wang et al. 2017). A recent study demonstrated that Drp1 acetylation induced by cardiac ischemic stress increased myocardial cell death and cardiac dysfunction(Manning et al. 2019). However, the role of GCN5L1 in the response of the brain to ischemia/hypoxia is currently unknown. Additionally, activation of AMPK, as the cellular energy sensor, by small-molecule activators, in the absence of mitochondrial damage, is sufficient to induce mitochondrial fission(Toyama et al. 2016), and its activation results in increased localization of Drp1 at the mitochondria, thus promoting Drp1-mediated mitochondrial fission(Herzig & Shaw. 2018; Wu et al. 2022). Although these observations demonstrated that AMPK is a crucial and direct regulator of mitochondrial dynamics, and that its activation is required for the fragmentation of mitochondria, the causal relationship between AMPK activation and Drp1 acetylation in cerebral ischemia still need further exploration.

In this study, we investigated the role and underlying mechanism of Drp1 acetylation in cerebral ischemia-induced mitochondrial fission in vivo and in vitro, and explored the upstream signaling regulating this process. Our results identify a previously unrecognized CDK5-AMPK-GCN5L1 pathway that mediates Drp1 acetylation and mitochondrial fission under cerebral ischemia/hypoxia conditions. Targeting this newly identified pathway may be a novel therapeutic option for the treatment of ischemic stroke.

## Materials and methods

### Cell culture, OGD and drug treatment

Neuro-2a cells and SH-SY5Y cells, procured from the American Type Culture Collection (ATCC), were cultured in Dulbecco’s Modified Eagle’s Medium (DMEM) (Gibco) supported by 10% fetal bovine serum (FBS) (BIOEXPLORER LifeSciences, BS1612-105) and antibiotics (100 µg/mL streptomycin and 100 U/mL penicillin). The cells were incubated at 37 °C in 5% CO_2_ atmosphere. For the induction of oxygen-glucose deprivation (OGD), cells were exposed to a hypoxic environment (94% N_2_, 1% O_2_, 5% CO_2_) with glucose-free DMEM mediums (Gibco), simulating an in vitro model of cerebral permanent ischemia. Compound C (inhibitor of AMPK) (5 µM, MCE, HY-13418 A) was acquired from MCE and dissolved in DMSO. The inhibitor was pre-treated for 20 h and oxygen-glucose deprivation for 4 h.

### Animal study

Male C57BL/6J mice, aged eight weeks and weighing 21–25 g, were procured from Cyagen and maintained under specific pathogen-free conditions as per the guidelines of the Animal Ethics Association at Hebei Medical University. Both cdk5 heterozygous knockout (cdk5^−/−^) and wild-type (WT) mice were employed to establish the permanent distal middle cerebral artery occlusion (dMCAO) model. Anesthesia was initiated using a 3.5% halothane in a 70% nitrous oxide and oxygen mixture. The procedural details of dMCAO have been previously elucidated. Using an operating microscope, a midline neck incision was performed to expose and ligate the right common carotid artery (CCA). Subsequent to that, a small hole was created in the skull using a high-speed dental drill to expose the right middle cerebral artery (MCA). Finally, the right side of the cortical branch of the MCA was electrocoagulated using a cauterizer (Bovie, USA) without causing damage to the brain surface. The temporal lobe area of dMCAO and sham mice was used for analysis. Mice in the sham group underwent isolation of the CCA without occlusion. A small hole was then created to expose the distal MCA, but coagulation was not performed. Compound C was acquired from MCE and dissolved in a mixture comprising 50% PEG300 and 50% 0.9% saline. The mice were intraperitoneally injected with Compound C (20 mg/kg) for 3 d before dMCAO and 3 d following dMCAO.

### Transfection

Neuro-2a cells were transfected with si-RNA using RNAFit and recombinant plasmids using Lipofectamine2000 (Thermo Fisher, USA), as the manufacturer’s protocol. Cells were seeded in 6-well plates and transfected 24 h later. Post-transfection, the medium was replaced with standard culture medium after 24 h. Western blot analysis and qRT-PCR were employed to assess the expression levels of GCN5L1 protein and mRNA.

### Cerebral infarction volume

Brain slices were obtained at 2-mm intervals from the previous halogen point and subjected to histochemical staining with 2% 2,3,5-triphenyltetrazolium chloride (TTC) at 37 ℃ for 20 min. The stained sections were then utilized for the measurement of infarct volume. The cerebral infract volume was quantified using Image J software. The infarct volumes were calculated as follows: percentage hemisphere lesion volume (% HLV) = {[total infarct volume – (volume of intact ipsilateral hemisphere–volume of intact contralateral hemisphere)]/ contralateral hemisphere volume} × 100%.

### Mitochondrial morphology

Mitochondrial structures within Neuro-2a and SH-SY5Y cell lines were fluorescently labeled using Mito-Tracker Red at a concentration of 50 nM, incubated at 37 ℃ for 30 min. Confocal microscopy was employed to visualize the labeled mitochondria. Quantification of mitochondrial morphology/mitochondrial area was measured by thresholding the MitoTracker fluorescence in ImageJ and dividing by the whole cell area(Dagda et al. 2009; Hemel et al. 2021). The fissed mitochondria have a smaller area than that of fused mitochondria.

### Mitochondrial ROS

Mitochondrial ROS (mtROS) levels were measured based on the fluorescence intensity derived from MitoSOX (MCE, HY-D1055) live-cell imaging, conducted using a 5 µmol/L solution for 10 min at 37 °C. The red fluorescence emitted by MitoSOX in Neuro-2a cells was detected with fluorescence microscope and flow cytometer. Frozen sections of brain tissues and in each group (5-µm-thick) were prepared and incubated with MitoSOX (5µM) for 30 min in the dark at 37 °C. The sections were observed using a fluorescence microscope (Leica, Germany). Ten fields were randomly selected from each sample.

### Immunohistochemical staining

For immunohistochemical staining, brain tissues were removed and immediately immersed in 4% paraformaldehyde for 48 h and then dehydrated in a graded series of alcohols and embedded in paraffin. Brain Sect. (4 μm thick) were blocked in 3% H_2_O_2_ to eliminate endogenous peroxidase activity and 5% normal goat serum, and then incubated overnight with anti-rabbit Drp1 (1:1000, Abcam), p-Drp1 (1:200, Cell Signaling Technology), p-AMPK (1:200, Cell Signaling Technology), CDK5 (1:500, Abcam), and GCN5L1 (1:500, Proteinch) rabbit polyclonal antibodies in PBS overnight at 4 °C. After a PBS wash, the sections were incubated with secondary antibody at 37 °C for 30 min. Subsequent staining was performed following a DAB (Zhongshan Goldenbridge Biotechnology, Beijing, China) immunostaining protocol.

### Immunofluorescence staining

Post-OGD and drug pretreatment, Neuro-2a cells were fixed with 4% paraformaldehyde for 20 min and permeabilized with 0.5% Triton X-100 for 5 min, repeated three times, following PBS washes. Blocking was carried out using goat serum for 30 min, after which cells were incubated with primary antibodies against Drp1 (1:150) (Abcam, ab9787) and GCN5L1 (1:100) (Proteintech, 19687-1-AP) at 4°C overnight. Cells were then washed and subjected to a secondary permeabilization step with 0.5% Triton X-100, followed by counterstaining with 4’,6-diamidino-2-phenylindole (DAPI) (SouthernBiotech, 0100- 20). This process was replicated for three independent experiments, and the Images were captured using fluorescence microscope (Leica DM6000B, Germany). Co-localization quantitation analysis of GCN5L1 and Drp1 was made by fluorescence integrated density measurements using Image J software (Hu et al. 2024; O’Brien et al. 2015).

### TUNEL staining

Following fixation and paraffin embedding, sections were deparaffinized and rehydrated. Protease K (20 µg/ml) was conducted at 20–37℃ for 15–30 min, devoid of DNase, followed by three PBS washes. TUNEL assay solution (Vazyme, A112) was prepared by combining TdT enzyme, fluorescent labeling solution, and detection solution. Samples were incubated with this solution at 37 °C for 60 min in the absence of light, followed by additional PBS washes. Finally, sections were mounted with DAPI and analyzed under a fluorescence microscope.

### Cell apoptosis

Annexin V/PI staining (BioLegend, 640932) was used to find cell apoptosis by flow cytometry. In brief, cells were seeded in 6-well plates. After treatment, cells were collected, washed with PBS twice. Cells were then incubated with Annexin V-FITC and PI at room temperature in the dark for 30 min. Flow cytometry analysis was conducted on a flow cytometer (Agilent) and data were analyzed using FlowJo V10 (v.10.0.7r2).

### Quantitative real-time polymerase chain reaction

Total RNA was extracted and then followed by two-step RT-PCR protocol using the E.Z.N.A. Total RNA Kit II (Omega, R6934-02). Specific primers targeting Drp1, GCN5L1, CDK5, AMPK and 18s rRNA were used as follows: Drp1 forward primer, 5′-GCCTCAGATCGTCGTAGTGG-3′, Drp1 reverse primer, 5′- AACAAATCCTAGCACCACGCAT − 3′, GCN5L1 forward primer 5′- CTGTCCCGCCTGCTCAAAGAAC − 3′, and GCN5L1 reverse primer, 5′-CCATTCCAATCCACTGGCCTGTC-3′, CDK5 forward primer, 5′-TTCATGATGTCCTGCATAGTGAC-3′, CDK5 reverse primer,5′-CTTATAGTCTGGCAGCTTGGTCA-3′, AMPK forward primer, 5′-TTGAAACCTGAAAATGTCCTGCT-3′, AMPK reverse primer, 5′-GGTGAGCCACAACTTGTTCTT-3′, 18s rRNA forward primer, 5′-CCATCCAATCGGTAGTAGCG-3′, 18s rRNA reverse primer, 5′- GTAACCCGTTGAACCCCATT-3′. qRT-PCR for Drp1, GCN5L1, CDK5, AMPK, 18s rRNA was performed after cDNA-synthesis at 60 °C for 30 min and a denaturation at 95 °C for 2 min. qRT-PCR amplification was performed by 40 cycles, each with a denaturation for 10 min at 95 °C, annealing for 10 s at 95 °C and elongation for 30 s at 60 °C. The final extension of the qRT-PCR products was performed at 4 °C for 10 min.

### Western blotting

Cells and tissue proteins were obtained using a cold lysis buffer containing PMSF and RIPA in a 1:100 ratio. Protein concentrations were determined using the BCA kit from Beyotime. For western-blotting, proteins were separated by SDS-PAGE and transferred onto polyvinylidenefluoride (PVDF) (Millipore) membranes. After incubated with 5% skimmed milk, the membranes were fixed with the anti-rabbit Drp1 (1:1000) (Abcam, ab9787), p-Drp1 (1:1000) (Cell Signaling Technology, 4494), GCN5L1 (1:500) (Proteintech, 19687-1-AP), CDK5(1:1000) (Abcam, ab40773), AMPK (1:1000) (Cell Signaling Technology, 5831), p-AMPK (1:1000) (Cell Signaling Technology, 2535) and β-actin (1:10000) (Abcam, ab198991) at 4 °C overnight and then incubated with appropriate secondary antibody for 1 h at room temperature. Detection was performed using an ECL system. Band intensity was quantitated using Image J software (NIH), and values were normalized to the intensities of the β-actin signal that served as a loading control.

### Co-immunoprecipitation (Co-IP)

Co-immunoprecipitation (Co-IP) was conducted in accordance with the Protein A/G Magnetic Beads IP Kit instructions. Protein A/G magnetic beads (MCE, HY-K0202) were washed three times in PBST and incubated with the targeted antibody in PBST for 30 min at room temperature to prepare antibody-conjugated immunomagnetic beads. After washing, cell lysate supernatants were combined with the beads to form an immunomagnetic beads-antibody-antigen complex. Following additional washes, the complex was resuspended in loading buffer for subsequent western blot analysis of endogenous protein interactions.

### Statistical analysis

Data were analyzed using GraphPad prism9 software. For continuous variables, the student *t*-test or Mann-Whitney *U* test was used for the comparison between the two groups. For the comparison of > 2 groups, one-way ANOVA with Dunnett’s post hoc correction. Values of *P* < 0.05 were considered statistically significant and denoted with 1, 2, 3, or 4 asterisks when < 0.05, 0.01, 0.005, or 0.0001, respectively.

## Results

### Ischemia/hypoxia contributes to apoptosis in OGD-treated neuronal cells and the ischemic brain tissues through inducing mitochondrial fission and mtROS production

**Fig. 1:**
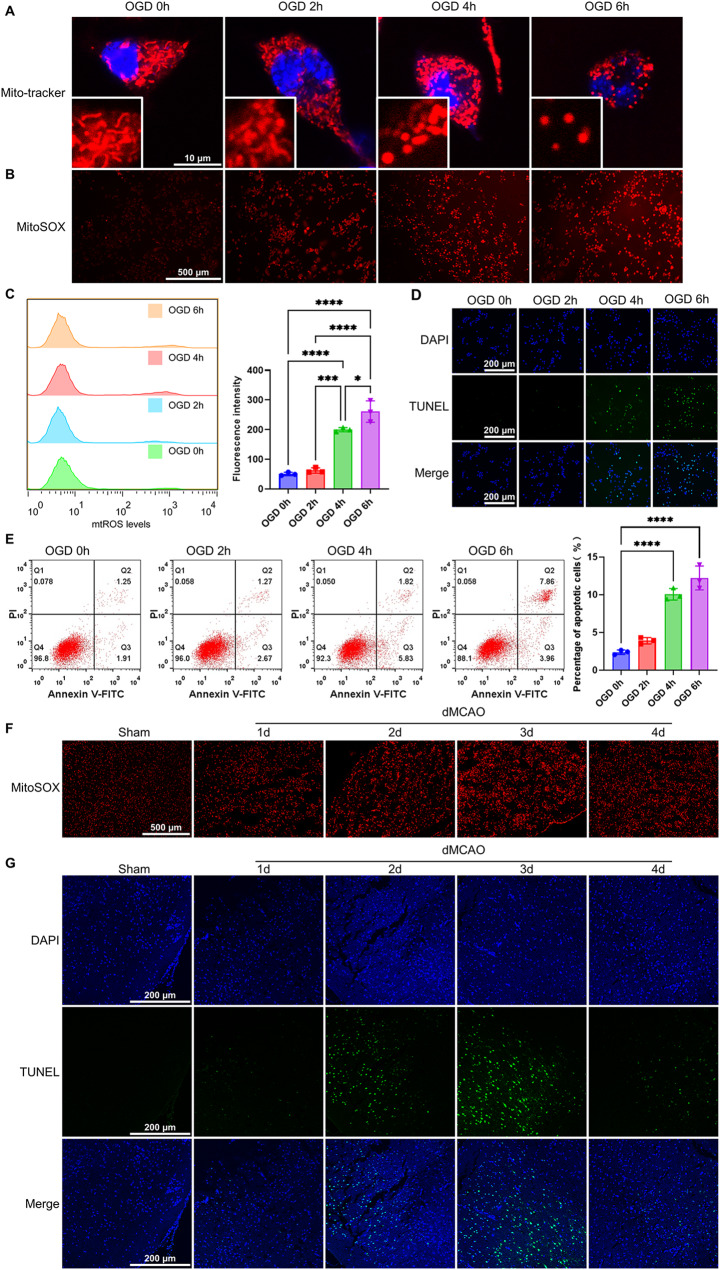
Ischemia/hypoxia induces mitochondrial fission, mtROS production and apoptosis in neuronal cells and brain tissues. (**A**) Mitochondrial morphology was visualized by 50 nM MitoTracker Red staining and observed by confocal microscopy in Neuro-2a cells treated with OGD for different times. Scale bars represent 10 μm (*n*=3). (**B**) Fluorescence images of MitoSOX-stained Neuro-2a cells treated as in (**A**). Scale bars represent 500 μm (*n*=4). (**C**) mtROS levels were analyzed in OGD-treated Neuro-2a cells by flow cytometry. Bar graphs show the mtROS levels measured based on fluorescence intensity (*n*=3). (**D**) TUNEL staining detected apoptosis in Neuro-2a cells treated with OGD for different times. Scale bars represent 200 μm (*n*=3). (**E**) Cell apoptosis analyzed by Annexin V-FITC/PI staining in OGD-treated cells for different times. Bar graphs show the percentage of apoptotic cells (*n*=3). (**F**) Fluorescence images of MitoSOX staining in brain sections of dMCAO and control mice. Scale bars represent 500 μm (*n*=3) (**G**) TUNEL staining detected apoptosis in brain sections of dMCAO and control mice. Scale bars represent 200 μm. Data are represented as mean ± SD, *P<0.05, ***P<0.005, ****P<0.0001, *P*-value was determined by one-way ANOVA with Dunnett’s post hoc correction

Because mitochondrial damage is one of the hallmarks of ischemic stroke and contributes to the pathology of ischemia/hypoxia (He et al. 2020), we first examined the effect of oxygen-glucose deprivation (OGD) on mitochondrial morphology in cultured Neuro-2a cells. Mitochondrial morphology was visualized in Neuro-2a cells treated with OGD for different times by immunofluorescent staining of Mitotracker red. The results showed that the mitochondria exist as network-like structures or rod-like shape (fused mitochondria) in the cells treated with OGD for 0 and 2 h, but when Neuro-2a cells were exposed to OGD for 4 and 6 h, the mitochondria became fragmented structures or punctate structures (fissed mitochondria) (Fig. 1A and Supplementary Fig. 1A). Similar results were observed in OGD-treated SH-SY5Y cells (Supplementary Fig. 1B and C). Mitochondrial fission is associated with mitochondrial ROS (mtROS) production (Shi et al. 2018), we next used MitoSOX staining to evaluate mtROS and found that mtROS levels were elevated with increasing of OGD treatment time (Fig. 1B and C), in parallel with the mitochondrial fission. Further, TUNEL staining and flow cytometry were used to detect OGD-induced apoptosis. As expected, increased mitochondrial fission and mtROS accumulation were accompanied by the increased apoptosis (Fig. 1D and E). These findings indicate that OGD treatment leads to Neuro-2a cell apoptosis by promoting mitochondrial fission and mtROS production.

To corroborate the result of the in vitro experiment, we established a mouse model of distal middle cerebral artery occlusion (dMCAO) and confirmed the successful establishment of the dMCAO model by TTC staining showing an extensive ischemia in the cerebral cortex (Supplementary Fig. 1D). MitoSOX and TUNEL staining showed that ischemic brain injury induced by dMCAO significantly enhanced mtROS production and cell apoptosis after 3 days of dMCAO (Fig. 1F and G). These results suggest that ischemia/hypoxia elicited by OGD and dMCAO promote mitochondrial fission in the neuronal cells, which subsequently leads to mtROS accumulation and cell apoptosis.

### The expression of Drp1 and GCN5L1 is up-regulated in OGD-treated neuronal cells and in the ischemic brain tissues

**Fig. 2:**
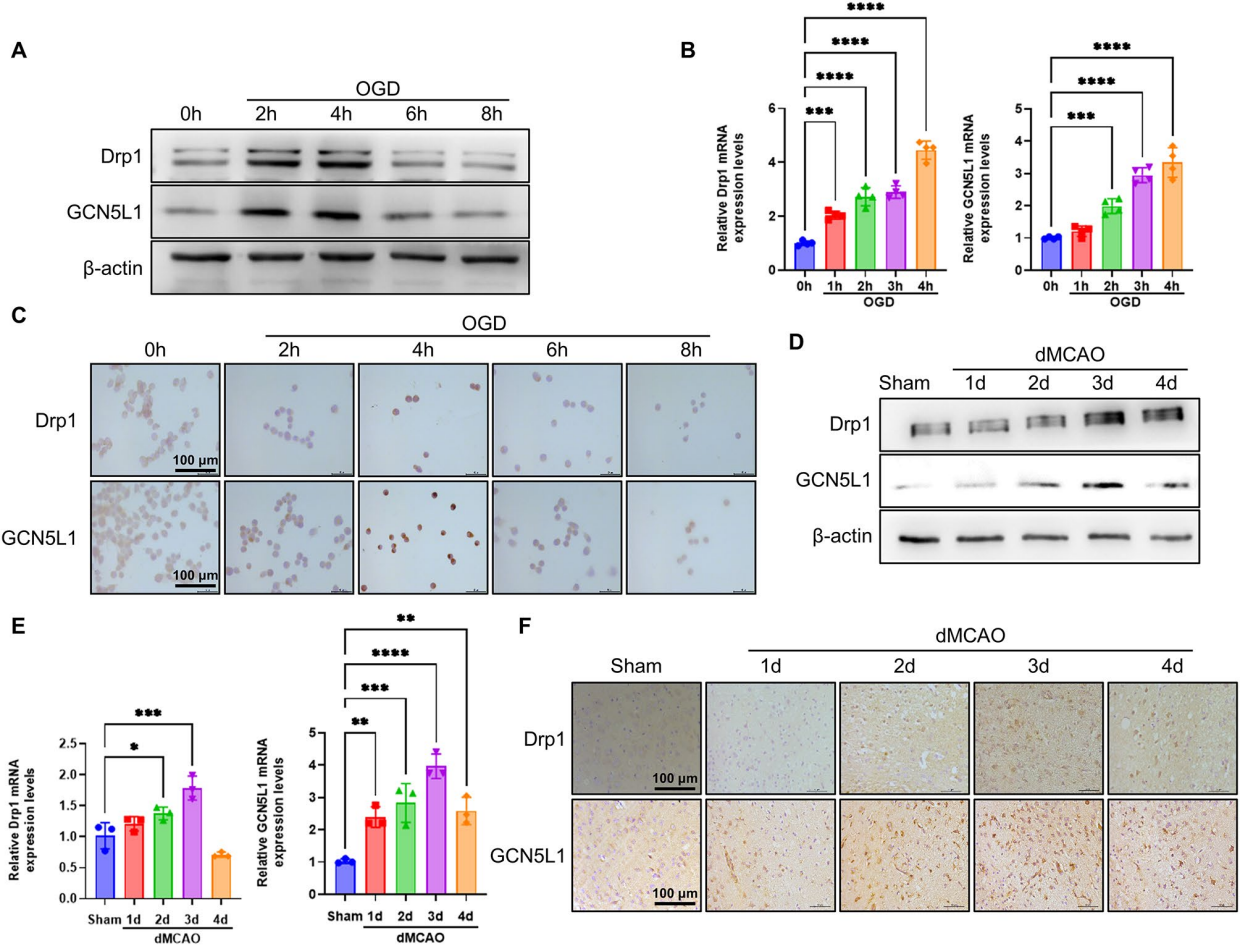
Drp1 and GCN5L1 are upregulated in OGD-treated neuronal cells and the ischemic brain tissues induced by dMCAO. (**A**) Western blot analysis of Drp1 and GCN5L1 in Neuro-2a cells treated with OGD for different times (*n*=6). (**B**) mRNA levels of Drp1 and GCN5L1 were determined by qRT-PCR in Neuro-2a cells treated with OGD for different times (*n*=4). (**C**) Immunohistochemistry staining for Drp1 and GCN5L1 in Neuro-2a cells treated with OGD for different times. Scale bars represent 100 μm (*n*=5). (**D**) Western blot analysis of Drp1 and GCN5L1 in brain tissues of dMCAO and control mice (*n*=6). (**E**) mRNA levels of Drp1 and GCN5L1 were determined by qRT-PCR in brain tissues of dMCAO and control mice (*n*=3). (**F**) Immunohistochemistry staining for Drp1 and GCN5L1 in brain sections of dMCAO and control mice. Scale bars represent 100 μm (*n*=5). Data are represented as mean ± SD, *P<0.05, **P<0.01, ***P<0.005, ****P<0.0001, *P*-value was determined by one-way ANOVA with Dunnett’s post hoc correction

Mitochondrial fission is known to be mediated by dynamin-related protein 1 (Drp1), whose activity is up-regulated by acetylation mediated by mitochondrial acetyltransferase GCN5L1(Wang et al. 2017). Therefore, we wanted to know whether ischemia/hypoxia-induced mitochondrial fission is relevant to the alteration of Drp1 and GCN5L1 expression. We found that the expression of Drp1 and GCN5L1 was significantly elevated 2 h after OGD treatment in Neuro-2a cells, with a peak at 4 h and later gradually reduced to the baseline level within 8 h (Fig. 2A and Supplementary Fig. 2A, B). Consistently, the level of Drp1 and GCN5L1 mRNA showed similar trends to that of their protein levels, as determined by qRT-PCR (Fig. 2B). Further, immunohistochemistry staining for Drp1 and GCN5L1 also showed the similar results as Western blot and qRT-PCR analysis, showing that Drp1 and GCN5L1 proteins were obviously increased in Neuro-2a cells treated with OGD for 4 h (Fig. 2C). In further studies, we determined the expression of Drp1 and GCN5L1 in ischemic brain tissues induced by dMCAO and found that these two proteins were significantly up-regulated at 3 days after dMCAO at both mRNA and protein levels, as shown by Western blot and qRT-PCR analysis (Fig. 2D, E and Supplementary Fig. 2C, D). And a similar result was also obtained by immunostaining for Drp1 and GCN5L1, showing that Drp1 and GCN5L1 expression was markedly increased in ischemic brain tissues 3 days post-dMCAO compared with that in sham-operation (Fig. 2F). These data suggest that the increased Drp1 and GCN5L1 expression may be responsible for ischemia/hypoxia-induced mitochondrial fission in neuronal cells.

### Ischemia/hypoxia facilitates Drp1 interaction with GCN5L1, subsequently leading to Drp1 acetylation

**Fig. 3:**
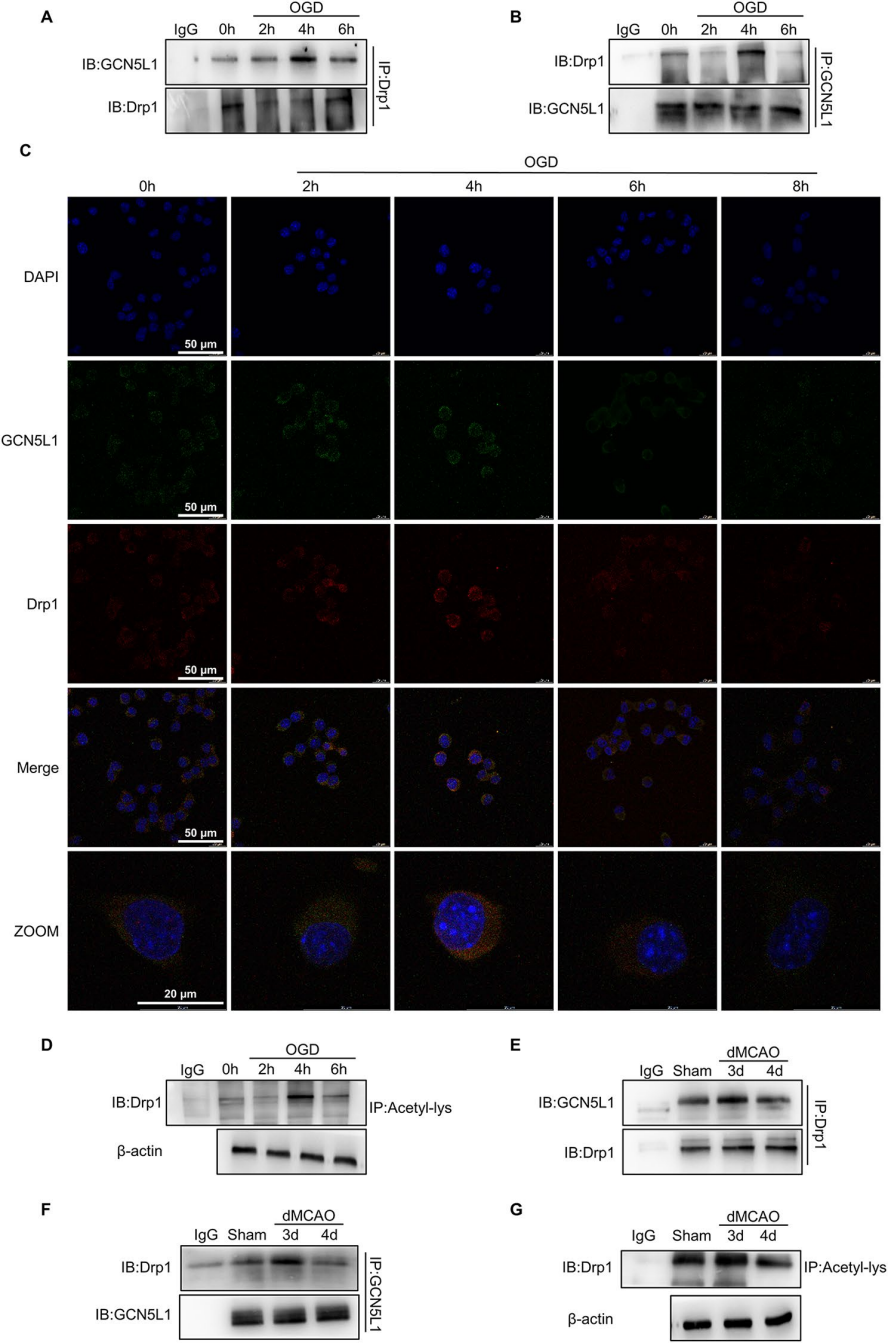
Ischemia/hypoxia facilitates Drp1 interaction with GCN5L1 and leads to Drp1 acetylation. (**A**-**B**) Interaction between Drp1 and GCN5L1 was detected by immunoprecipitates (IP) in Neuro-2a cells using anti-GCN5L1 for IP and anti-Drp1 for western blotting, as well as with anti-Drp1 for IP and anti-GCN5L1 for western blotting (*n*=3). (**C**) Co-localization between Drp1 and GCN5L1 was detected by immunofluorescence staining of Drp1 (red), GCN5L1 (green), and DAPI (blue) in neuro-2a cells treated with OGD for different times. Scale bars represent 50 μm and 20 μm (*n*=3). (**D**) Acetylated Drp1 was analyzed in Neuro-2a cells treated with OGD for different times by IP with anti-acetyl lysine antibody and western blotting with anti-Drp1 (*n*=3). (**E**-**F**) Interaction between Drp1 and GCN5L1 was detected by IP in brain tissues of dMCAO and control mice using anti-GCN5L1 for IP and anti-Drp1 for western blotting as well as with anti-Drp1 for IP and anti-GCN5L1 for western blotting (*n*=3). (**G**) Acetylated Drp1 was analyzed in brain tissues of dMCAO and control mice by IP with anti-acetyl lysine antibody and western blotting with anti-Drp1 (*n*=3)

The above studies suggest that ischemia/hypoxia up-regulated Drp1 and GCN5L1 expression, and previous studies identified acetylation as a novel post-translational Drp1 modification that regulates its activity(Hu et al. 2020). We therefore wondered whether GCN5L1 acetylates Drp1 by interacting with each other. To this end, we conducted a co-immunoprecipitation experiment (Co-IP) and found the level of GCN5L1 co-immunoprecipitated by anti-Drp1 antibody was markedly increased in Neuro-2a cells treated with OGD for 4 h. Likewise, a similar result was obtained by reciprocal immunoprecipitation with anti-GCN5L1 antibody (Fig. 3A, B and Supplementary Fig. 3A, B). Furthermore, confocal immunofluorescence staining showed an increased co-localization between GCN5L1 and Drp1 in the cytoplasm of Neuro-2a cells exposed to OGD for 4 h (Fig. 3C and Supplementary Fig. 3C). These observations indicate that there exists an interaction between GCN5L1 and Drp1 and their interaction was strengthened with treatment of OGD in neuronal cells. As expected, an obvious increase in Drp1 acetylation was observed in OGD-treated Neuro-2a cells for 4 h along with increased binding of GCN5L1 to Drp1 (Fig. 3D and Supplementary Fig. 3D). To assess translational relevance of these findings, we also examined the interaction of GCN5L1 with Drp1 and Drp1 acetylation in mice undergoing dMCAO surgery. The results showed that the association of GCN5L1 with Drp1 and thus Drp1 acetylation were significantly enhanced at 3 days after dMCAO surgery, consistent with observations of the in vitro experiment (Fig. 3E-G and Supplementary Fig. 3E-G). Collectively, these findings suggest that GCN5L1 interacts with and acetylates Drp1 in OGD-treated neuronal cells as well as in the ischemic brain tissues.

### Knockdown or overexpression of GCN5L1 attenuates or enhances mitochondrial fission in neuronal cells

**Fig. 4:**
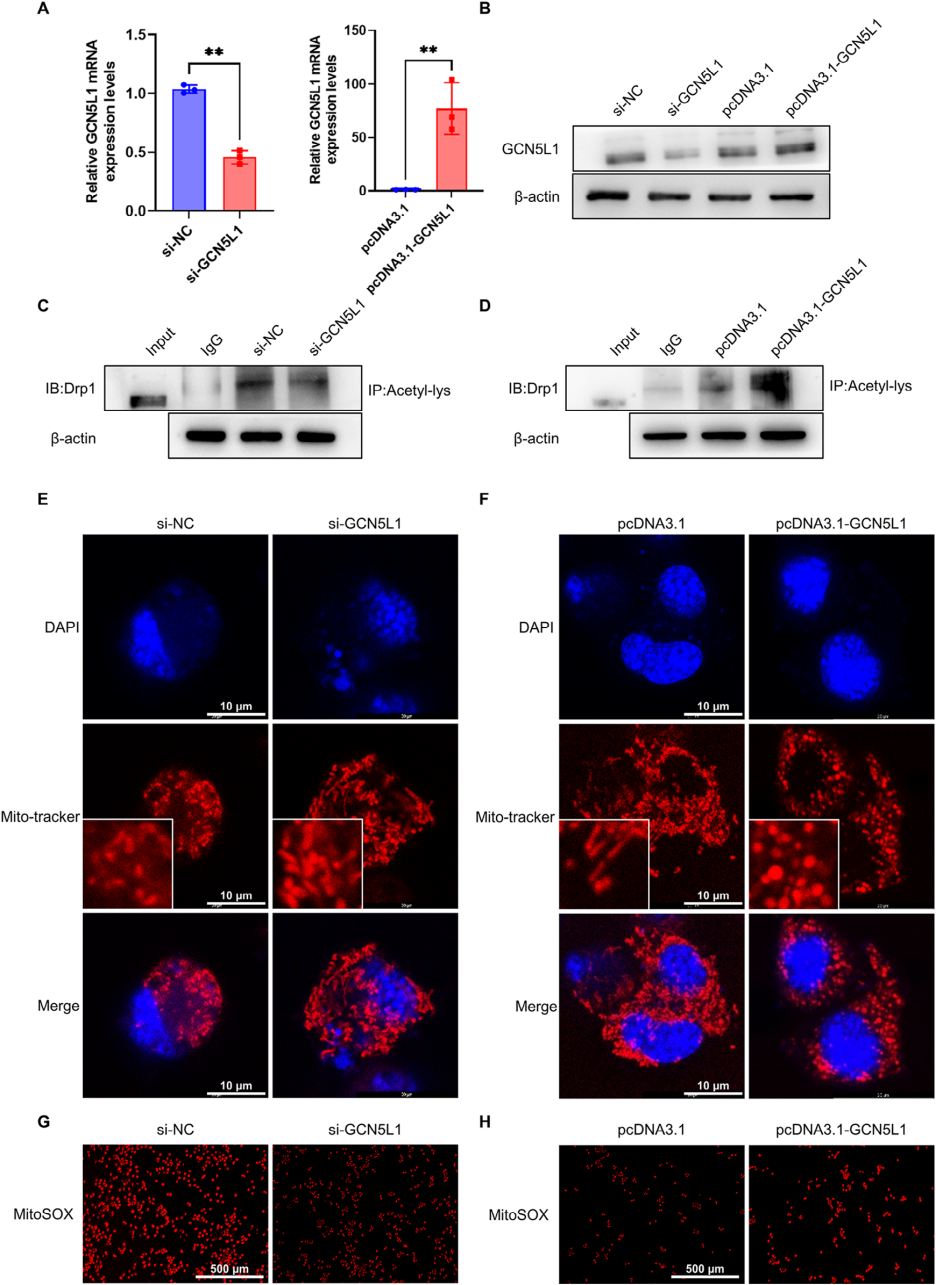
Knockdown or overexpression of GCN5L1 attenuates or enhances mitochondrial fission in neuronal cells. (**A**) qRT-PCR detected the expression of GCN5L1 mRNA in si-GCN5L1 or pcDNA3.1-GCN5L1-transfected Neuro-2a cells (*n*=3). (**B**) Western blotting detected the expression of GCN5L1 in Neuro-2a cells transfected with si-GCN5L1 or pcDNA3.1-GCN5L1 (*n*=3). (**C**-**D**) Acetylated Drp1 was analyzed by IP with anti-acetyl lysine antibody and western blotting with anti-Drp1 after Neuro-2a cells were transfected with si-GCN5L1 or pcDNA3.1-GCN5L1 (*n*=3). (**E**-**F**) Mitochondrial morphology was evaluated by Mito-Tracker Red staining and confocal microscope in Neuro-2a cells transfected with si-GCN5L1 or pcDNA3.1-GCN5L1 and then treated with OGD. Scale bars represent 10 μm (*n*=3). (**G**-**H**) Immunofluorescent staining of MitoSOX in Neuro-2a cells transfected with si-GCN5L1 or pcDNA3.1-GCN5L1. Scale bars represent 500 μm (*n*=5). Data are represented as mean ± SD, ** *P*<0.01, *P*-value was determined by student t-test

Given that Drp1 acetylation mediated by GCN5L1 exerts a key role in the mitochondrial fission induced by ischemia/hypoxia, we manipulated GCN5L1 expression in Neuro-2a cells by transfection with either si-GCN5L1 or pcDNA3.1- GCN5L1 and their respective controls and examined the acetylation of Drp1. Figure 4A, B and Supplementary Fig. 4A show successful knockdown or overexpression of GCN5L1 mRNA and protein in Neuro-2a cells. We found that the silencing GCN5L1 expression by its specific siRNA significantly reduced the level of Drp1 acetylation, while its overexpression mediated by GCN5L1-expressing plasmid exerted the opposite effect (Fig. 4C, D and Supplementary Fig. 4B). These data clearly suggest that GCN5L1 mediates acetylation of Drp1 through interacting with Drp1. In further experiments, we determined the effects GCN5L1-mediated acetylation of Drp1 on mitochondrial morphology. Immunofluorescent staining of Neuro-2a cells using Mito-Tracker revealed that GCN5L1 knockdown obviously facilitated the formation of network-like mitochondria or rod-like shape (fused mitochondria). In contrast, fragmented or punctate mitochondria (fissed mitochondria) were increased in GCN5L1-overexpressing cells (Fig. 4E, F and Supplementary Fig. 4C, D). Correspondingly, the mtROS production was also reduced obviously in GCN5L1-depleted Neuro-2a cells or increased in GCN5L1-overexpressing cells (Fig. 4G and H). Taken together, these data demonstrate that GCN5L1 plays an important role in ischemia/hypoxia-induced mitochondrial fission through acetylating Drp1 in neuronal cells.

### Cdk5 deficiency protects brain tissue against ischemic injury in mice via attenuating Drp1 and GCN5L1 expression and Drp1 acetylation

**Fig. 5:**
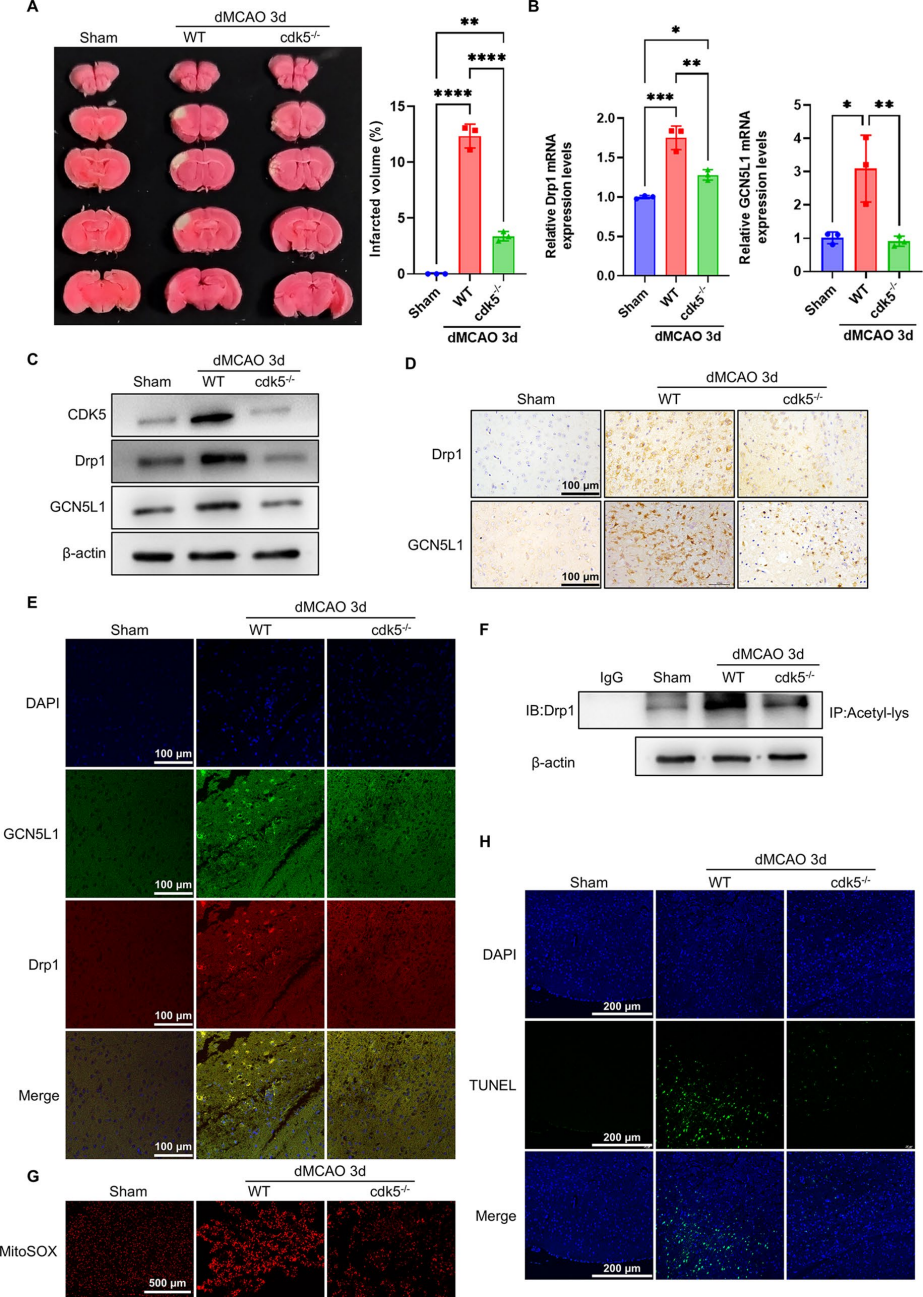
cdk5 deficiency protects brain tissues against ischemic injury in mice. (**A**) Representative TTC-stained brain sections and quantitative analysis of infarct volume (*n*=3). (**B**) mRNA levels of Drp1 and GCN5L1 were determined by qRT-PCR in ischemic brain tissues of WT and cdk5 knockout mice (*n*=3). (**C**) Western blot analysis of CDK5, Drp1 and GCN5L1 in ischemic brain tissues of WT and cdk5 knockout mice (*n*=3). (**D**) Immunohistochemistry staining for Drp1 and GCN5L1 in brain sections of WT and cdk5 knockout mice. Scale bars represent 100 μm (*n*=3). (**E**) Co-localization between Drp1 and GCN5L1 was detected by immunofluorescence staining of GCN5L1 (green), Drp1 (red), and DAPI (blue) in brain tissues of WT and cdk5 knockout mice. Scale bars represent 100 μm (*n*=3). (**F**) Acetylated Drp1 was analyzed in brain tissues of WT and cdk5 knockout mice by IP with anti-acetyl lysine antibody and western blotting with anti-Drp1 (*n*=3). (**G**) Fluorescence images of MitoSOX staining in brain sections of WT and cdk5 knockout mice. Scale bars represent 500 μm (*n*=3). (**H**) TUNEL staining detected apoptosis in brain sections of WT and cdk5 knockout mice. Scale bars represent 200 μm (*n*=3). Data are represented as mean ± SD, * *P* <0.05, ** *P* <0.01, ***P<0.005, ****P<0.0001, *P*-value was determined by one-way ANOVA with Dunnett’s post hoc correction

Because CDK5 is known to play a significant role in the regulation of mitochondrial morphology(Cho et al. 2014; Meuer et al. 2007), we next investigated the potential link between CDK5, Drp1 and GCN5L1 during neuronal cell death induced by ischemia/hypoxia. As shown in Fig. 5A, global cdk5 knockout mice had a significantly decreased volume of the infarcted brain tissue after dMCAO surgery compared with that in WT mice. Further, we assessed whether the protective effect of cdk5 deficiency on cerebral ischemic injury is related to Drp1 and GCN5L1. qRT-PCR and Western blot analysis showed that the expression of Drp1 and GCN5L1 in the infarcted brain tissue of cdk5 knockout mice was significantly reduced 3 days after MCAO at both mRNA and protein levels compared with that in WT mice (Fig. 5B, C and Supplementary Fig. 5A-C). Similarly, the down-regulation of Drp1 and GCN5L1 expression in dMCAO-induced brain damage of Cdk5-deficient mice was also verified through immunohistochemical staining (Fig. 5D). These results suggest that cdk5 depletion protects brain tissue against ischemic injury induced by MCAO in mice via down-regulating Drp1 and GCN5L1 expression. In further experiments, we detected the effect of cdk5 deficiency on the interaction of GCN5L1 with Drp1 and Drp1 acetylation. As shown in Fig. 5E, F and Supplementary Fig. 5D, E, cdk5 knockout obviously declined Drp1 and GCN5L1 interaction and Drp1 acetylation in the infarcted brain tissue relative to dMCAO-operated WT mice. As expected, MitoSOX staining with FACS analysis showed that mtROS accumulation was decreased in the infarcted brain tissue of cdk5 deficient mice compared to that in WT mice (Fig. 5G). In parallel with alteration of mtROS level, cdk5 deficiency also reduced cell apoptosis in the infarcted brain tissue induced by dMCAO, as evidenced by TUNEL staining (Fig. 5H).

### Ischemia/hypoxia upregulates CDK5 expression and induces the phosphorylation of AMPK and Drp1 in OGD-treated neuronal cells and in the ischemic brain tissues

**Fig. 6:**
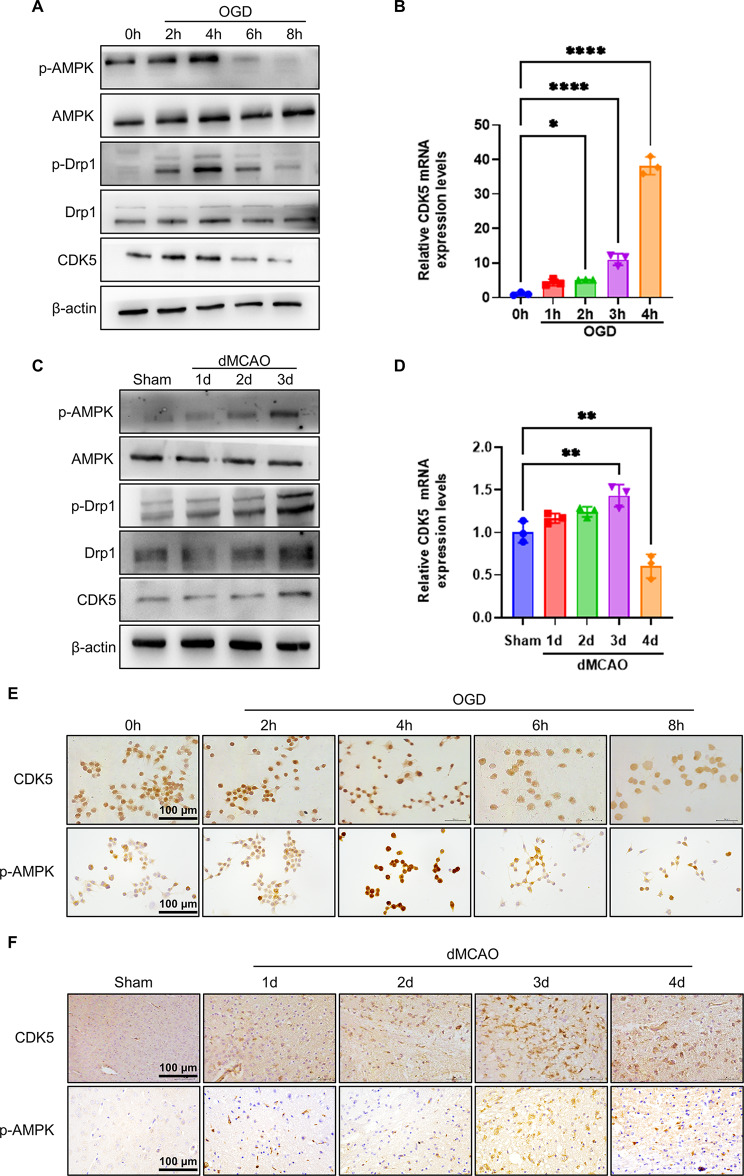
Ischemia/hypoxia upregulates CDK5 expression and induces the phosphorylation of AMPK and Drp1. (**A**) Western blot analysis of p-AMPK, AMPK, p-Drp1, Drp1 and CDK5 in Neuro-2a cells treated with OGD for different times (*n*=3). (**B**) mRNA levels of CDK5 were determined by qRT-PCR in Neuro-2a cells treated with OGD for different times (*n*=3). (**C**) Western blot analysis of p-AMPK, AMPK, p-Drp1, Drp1 and CDK5 in brain tissues of dMCAO and control mice (*n*=3). (**D**) mRNA levels of CDK5 were determined by qRT-PCR in brain tissues of dMCAO and control mice (*n*=3). (**E**) Immunohistochemistry staining for CDK5 and p-AMPK in Neuro-2a cells treated with OGD for different times. Scale bars represent 100 μm (*n*=5). (**F**) Immunohistochemistry staining for CDK5 and p-AMPK in brain sections of dMCAO and control mice. Scale bars represent 100 μm (*n*=5). Data are represented as mean ± SD, *P<0.05, **P<0.01, ****P<0.0001, *P*-value was determined by one-way ANOVA with Dunnett’s post hoc correction

Because recent studies indicate that CDK5 promotes mitochondrial fission via Drp1 phosphorylation at S616 in chronic ethanol exposure(Liu et al. 2022), and because AMPK activation results in increased neuron death during hypoxic/ischaemic brain injury(Jiang et al. 2018), we hypothesized that AMPK might participate in the regulation of CDK5 on Drp1 phosphorylation. To test this hypothesis, we examined the influence of hypoxia/ischaemia on CDK5 expression and the phosphorylation of AMPK and Drp1. As shown in Fig. 6A, B and Supplementary Fig. 6A-D, CDK5 expression was significantly up-regulated in Neuro-2a cells treated with OGD for 2 h, with a peak at 4 h, at both mRNA and protein levels. Consistently, Western blot and qRT-PCR analysis also showed a significant increase in CDK5 protein and mRNA in ischemic cerebral tissues 3 days after dMCAO (Fig. 6C, D and Supplementary Fig. 6E-H). In parallel to the changes in CDK5 expression levels, the phosphorylation of AMPK and Drp1 markedly increased, respectively, at 4 h after OGD and 3 days after dMCAO (Fig. 6A and C). Moreover, the alterations of CDK5 expression and AMPK phosphorylation were further validated via immunohistochemical staining in OGD-treated Neuro-2a cells as well as in the ischemic brain tissues induced by dMCAO (Fig. 6E and F). Collectively, these data imply that the phosphorylation of Drp1 is correlated with CDK5 up-regulation and AMPK activation by hypoxia/ischaemia in neuronal cells.

### Inhibition of AMPK suppresses Drp1 phosphorylation and interaction of GCN5L1 with Drp1, thus attenuating Drp1 acetylation and mitochondrial fission in OGD-treated neuronal cells

**Fig. 7:**
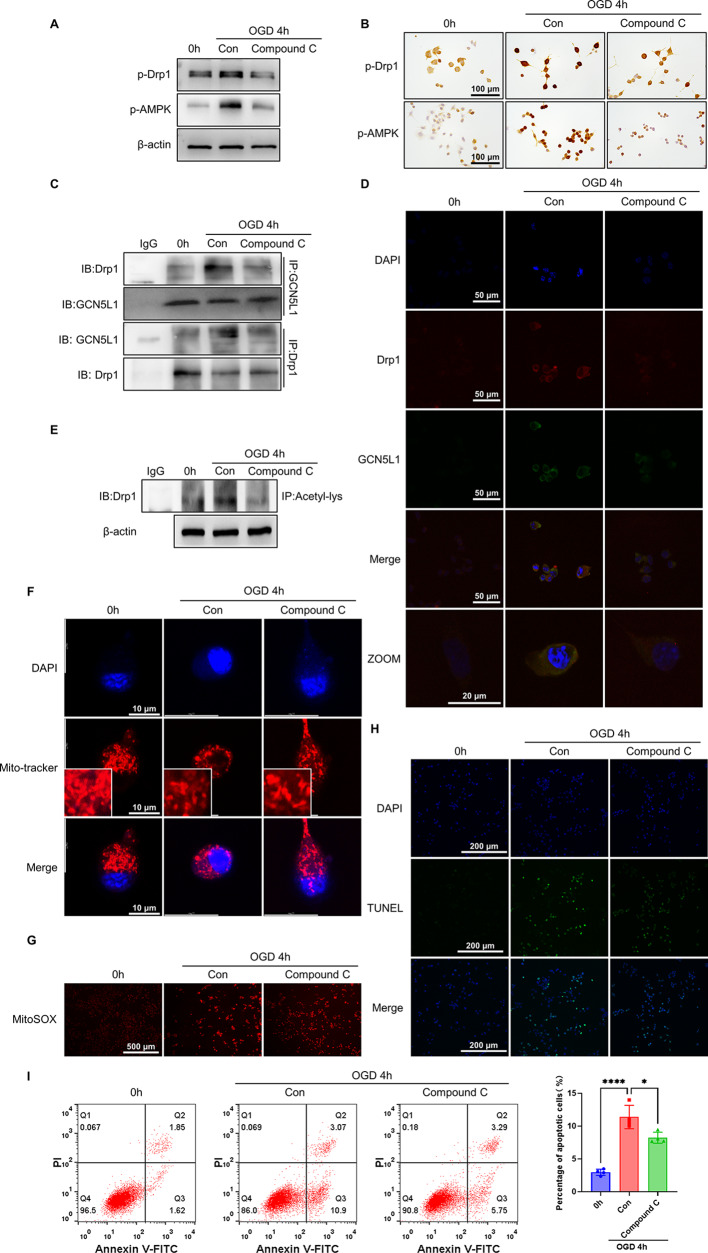
Inhibition of AMPK attenuates Drp1 acetylation and mitochondrial fission in OGD-treated neuronal cells. (**A**) Western blot analysis of p-Drp1 and p-AMPK in neuro-2a cells treated with OGD and Compound C (*n*=3). (**B**) Immunohistochemistry staining for p-Drp1 and p-AMPK in Neuro-2a cells treated with OGD and Compound C. Scale bars represent 100 μm (*n*=3). (**C**) Interaction between Drp1 and GCN5L1 was detected by IP in Neuro-2a cells treated with OGD and Compound C (*n*=3). (**D**) Co-localization between Drp1 and GCN5L1 was detected by immunofluorescence staining of Drp1 (red), GCN5L1 (green), and DAPI (blue) in Neuro-2a cells treated with OGD and Compound C. Scale bars represent 50 μm and 20 μm, respectively (*n*=3). (**E**) Acetylated Drp1 was analyzed in Neuro-2a cells treated with OGD and Compound C by IP with anti-acetyl lysine antibody and western blotting with anti-Drp1 (*n*=3). (**F**) Mitochondrial morphology was visualized by 50 nM Mito-Tracker Red staining and observed by confocal microscopy in Neuro-2a cells treated with OGD and Compound C. Scale bars represent 10 μm (*n*=3). (**G**) Fluorescence images of MitoSOX staining in Neuro-2a cells treated with OGD and Compound C. Scale bars represent 500 μm (*n*=3). (**H**) TUNEL staining detected apoptosis in Neuro-2a cells treated with OGD and Compound C. Scale bars represent 200 μm (*n*=3). (**I**) Cell apoptosis analyzed by Annexin V-FITC/PI staining after Neuro-2a cells were treated with OGD and Compound C. Bar graphs show the percentage of apoptotic cells (*n*=4). Data are represented as mean ± SD, *P<0.05, ****P<0.0001, *P*-value was determined by one-way ANOVA with Dunnett’s post hoc correction

To further explore the involvement of AMPK in hypoxia/ischaemia-induced Drp1 phosphorylation and mitochondrial fission, we treated Neuro-2a cells with Compound C, a selective inhibitor of AMPK, and examined Drp1 modification and mitochondrial morphology. Western blot and immunohistochemical staining revealed that the phosphorylation of AMPK and Drp1 induced by OGD was substantially down-regulated in Compound C-treated Neuro-2a cells compared with vehicle-treated cells (Fig. 7A, B and Supplementary Fig. 7A), indicating Drp1 is a downstream molecule of AMPK signaling. We then performed co-immunoprecipitation experiments to determine the effect of Compound C on the interaction between GCN5L1 and Drp1. The results showed that treating Neuro-2a cells with AMPK inhibitor robustly decreased the association of GCN5L1 with Drp1 compared to vehicle-treated cells, as evidenced by a reciprocal immunoprecipitation assay (Fig. 7C and Supplementary Fig. 7B). This observation was further validated by confocal immunofluorescence staining of GCN5L1 and Drp1 (Fig. 7D and Supplementary Fig. 7C). These findings suggest that Drp1 phosphorylation by AMPK signaling is necessary for the interaction of GCN5L1 with Drp1. As expected, inhibition of AMPK by Compound C also markedly reduced Drp1 acetylation in Neuro-2a cells treated with OGD for 4 h (Fig. 7E and Supplementary Fig. 7D). Further, we assessed the impact of Compound C on mitochondrial morphology, mtROS production, and cell apoptosis using the OGD-treated Neuro-2a cells. The results showed that the majority of mitochondria existed as punctate-like shape (fissed mitochondria) in the cells treated for 4 h with OGD. In contrast, the number of fragmented mitochondria was much lower in Compound C-treated cells than in vehicle-treated cells (Fig. 7F and Supplementary Fig. 7E). Also, mtROS was visualized in vehicle- and Compound C-treated Neuro-2a cells by immunofluorescent staining using MitoSOX Red, and an obvious decline of mtROS level in Compound C-treated cells was concomitant with a decrease in mitochondrial fission (Fig. 7G). Simultaneously, TUNEL staining and flow cytometry revealed that Compound C treatment significantly suppressed cell apoptosis in OGD-treated Neuro-2a cells (Fig. 7H and I). Taken together, these results suggest that inhibition of AMPK alleviates OGD-induced mitochondrial fission and neuronal apoptosis through repressing AMPK-mediated Drp1 phosphorylation and thus its interaction with GCN5L1.

### Inhibition of AMPK signal by Compound C protects brain tissue from ischemic damage in mouse models of dMCAO

**Fig. 8:**
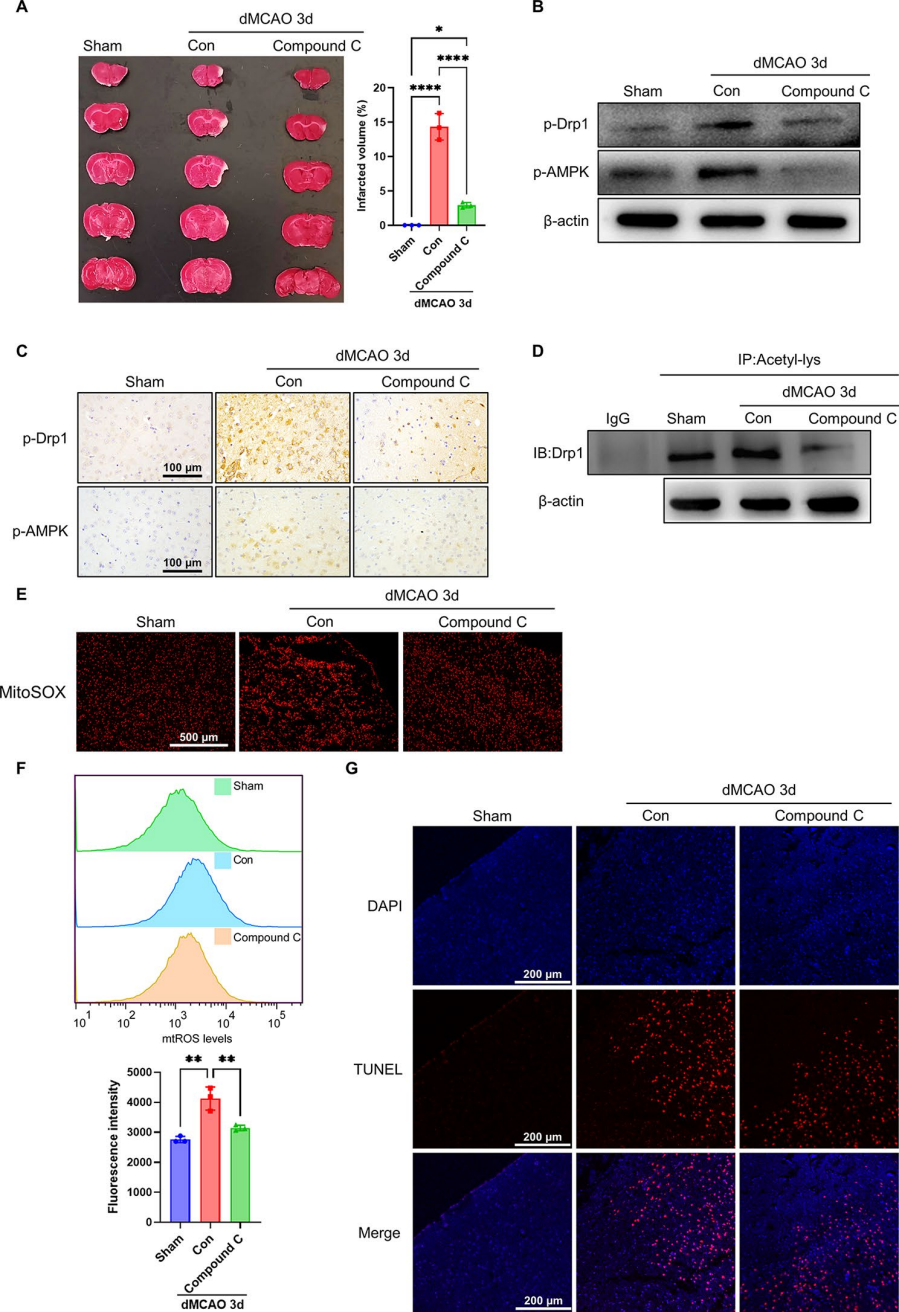
Inhibition of AMPK by Compound C protects brain tissues from ischemic damage in mouse models of dMCAO. (**A**) Representative TTC-stained brain sections and quantitative analysis of infarct volume (*n*=3). (**B**) Western blot analysis of p-Drp1 and p-AMPK in brain tissues of dMCAO and control mice treated with or without Compound C (*n*=3). (**C**) Immunohistochemistry staining for p-Drp1 and p-AMPK in brain tissues of dMCAO and control mice treated as in (**B**). Scale bars represent 100 μm (*n*=3). (**D**) Acetylated Drp1 was analyzed in brain tissues of dMCAO and control mice treated as in (**B**) by IP with anti-acetyl lysine antibody and western blotting with anti-Drp1 (*n*=3). (**E**) Fluorescence images of MitoSOX staining in brain sections of dMCAO and control mice treated as in (**B**). Scale bars represent 500 μm (*n*=3). (**F**) mtROS levels were analyzed by flow cytometry. Bar graphs show the mtROS levels measured based on fluorescence intensity (*n*=3). (**G**) TUNEL staining detected apoptosis in brain tissues of dMCAO and control mice treated as in (**B**). Scale bars represent 200 μm (*n*=3). Data are represented as mean ± SD, *P<0.05, **P<0.01, ****P<0.0001, *P*-value was determined by one-way ANOVA with Dunnett’s post hoc correction

To extend the above results obtained in vitro experiments using the cultured Neuro-2a cells, we utilized a mouse model of dMCAO to investigate the effect of AMPK inhibition on cerebral infarct volume. As shown in Fig. 8A, inhibition of AMPK by Compound C significantly reduced cerebral infarct sizes as determined by TTC staining. We then evaluated whether the protective effect of Compound C against cerebral ischemic injury is related to its regulation of Drp1 phosphorylation and acetylation. Western blot analysis and immunohistochemical staining showed a significant increase in AMPK and Drp1 phosphorylation in ischemic cerebral tissues 3 days after dMCAO, whereas these changes were blunted in the Compound C-treated mouse model of dMCAO (Fig. 8B, C and Supplementary Fig. 8A). Consistent with the inhibitory effect of Compound C on the phosphorylation of AMPK and Drp1, Compound C also significantly inhibited Drp1 acetylation induced by dMCAO (Fig. 8D and Supplementary Fig. 8B). Correspondingly, mtROS level measured by mitoSOX staining of the tissue sections as well as by FACS analysis of mitoSOX staining was robustly decreased in the ischemic brain tissues treated with Compound C (Fig. 8E and F). Likewise, treating MCAO mice with Compound C substantially diminished neuronal apoptosis in induced by cerebral ischemia compared with vehicle-treated mice (Fig. 8G). These findings were in agreement with what we observed in in vitro experiments, suggesting therapeutical significance of Compound C as a potential drug against cerebral ischemic injury.

## Discussion

Acetylation/deacetylation of mitochondrial proteins is a key regulatory mechanism of mitochondrial metabolism and function. The alteration in mitochondrial protein acetylation has been known to be involved in the pathogenesis of multiple human diseases including neurodegenerative and cardiovascular diseases, diabetes, cancer, and aging(Parodi-Rullán et al. 2018). Growing evidence has demonstrated that the level of mitochondrial protein acetylation is tightly regulated by two opposing enzymatic activities of mitochondrial acetyltransferase and NAD-dependent deacetylase sirtuin 3 (Sirt3), a major mitochondrial deacetylase, which requires NAD + for its deacetylase activity. Because the NAD + levels are significantly reduced following acute brain injury induced by ischemic insult as well as in mitochondria isolated from post-ischemic brain tissue(Klimova, Fearnow, Long, et al. 2020; Long et al. 2017), mitochondrial proteins show an excessive acetylation during the first 24 h of ischemia/reperfusion(Klimova, Fearnow, & Kristian. 2020b; Klimova, Fearnow, Long, et al. 2020). Of note, previous studies on mitochondrial protein acetylation have focused mostly on the relationship between the mitochondrial NAD + depletion and mitochondrial deacetylase activity, and the mitochondrial NAD + depletion has been regarded as the most important reason to cause hyperacetylation of mitochondrial proteins, which can result in altered mitochondrial metabolism and mitochondrial dynamics(Klimova, Long, & Kristian. 2019). Until recently, not much was known about how Drp1 is acetylated in ischemic/hypoxic brain tissues. In the current study, our results showed that Drp1 and GCN5L1 expression was significantly up-regulated in Neuro-2a cells treated with OGD for 4 h as well as in ischemic brain tissues 3 days post-dMCAO compared with their corresponding controls, which was concurrently accompanied by increased mitochondrial fission, mtROS production and cell apoptosis. More importantly, we revealed for the first time that GCN5L1 interacted with and acetylated Drp1 in OGD-treated neuronal cells and in the ischemic brain tissues. These observations were confirmed by further experiments showing that the knocking down GCN5L1 expression markedly reduced the level of Drp1 acetylation, while its overexpression enhanced Drp1 acetylation. Moreover, GCN5L1 knockdown promoted mitochondrial fission, whereas overexpression of GCN5L1 exerted the opposite effect. These findings clearly suggested that GCN5L1 plays a crucial role in ischemia/hypoxia-induced mitochondrial fission by mediating Drp1 acetylation in neuronal cells. Our study provides a novel pathophysiological mechanism linking mitochondrial dysfunction and ischemic/hypoxic brain injury through GCN5L1-mediated Drp1 acetylation.

GCN5L1 has previously been shown to regulate the acetylation of several mitochondrial fatty acid oxidation, glucose oxidation and electron transport chain proteins(Scott et al. 2012; Thapa et al. 2017, 2018). Increased GCN5L1 expression in response to a high fat diet (HFD) promoted increased lysine acetylation, resulting in the development of ROS-induced damage caused by nutrient excess, and the acetylation of cardiac mitochondrial proteins by GCN5L1 under HFD conditions may result in cardiac dysfunction(Thapa et al. 2020). Additionally, mitochondrial GCN5L1 was reported to modulate post-translational control of FoxO1, regulate gluconeogenesis and control metabolic pathways via mitochondrial ROS mediated ERK activation(Wang et al. 2017). However, it is not known whether GCN5L1 is operable in regulating mitochondrial dynamics-related proteins, or whether ischemia/hypoxia affects its regulation of Drp1 acetylation in brain tissues. To the best of our knowledge, this study was the first to find that GCN5L1 can acetylate mitochondrial dynamics-related protein Drp1 and that its up-regulation in the ischemic brain tissues leads to mitochondrial fission via promoting Drp1 acetylation.

Considering that acetylation of Drp1 requires its interaction with GCN5L1 and protein-protein interaction is most often regulated by phosphorylation of the interacting protein(Clokie et al. 2009), we explored the upstream signaling pathway regulating Drp1 phosphorylation and thus enhancing its interaction with GCN5L1. Although previous studies have shown that Drp1 can be phosphorylated by CDK1/cyclin B, ERK1/2, PKC, and CaMKII signaling in cardiomyocytes and the heart(Adaniya et al. 2019; Breitzig et al. 2018), it is still unclear whether AMPK, a central regulator of mitochondrial biogenesis, is responsible for the regulation of Drp1 phosphorylation under hypoxic/ischemic condition in the brain tissues. In this study, we found that the phosphorylation of AMPK and Drp1 was significantly induced at 4 h after OGD and 3 days after dMCAO. Notably, inhibition of AMPK by AMPK selective inhibitor Compound C obviously suppressed Drp1 phosphorylation, suggesting that Drp1 is a downstream molecule of AMPK signaling. Further, co-immunoprecipitation experiments and immunofluorescence staining demonstrated that AMPK inhibitor attenuated the interaction between GCN5L1 and Drp1 in OGD-treated Neuro-2a cells as well as in the ischemic brain tissues. These data suggest that Drp1 phosphorylation by AMPK signaling is required for the interaction of GCN5L1 with Drp1. Also, inhibition of AMPK by Compound C reduced ischemia/hypoxia-induced Drp1 acetylation and mitochondrial fission, thus alleviating neuronal damage and brain injury caused by ischemia/hypoxia. Consistent with our observation, a previous study found that activation of AMPK resulted in increased localization of Drp1 at the mitochondria, and this effect was dependent on the presence of the AMPK phosphorylation sites in another mitochondrial fission factor MFF(Toyama et al. 2016). Altogether, these data indicate that AMPK activation is responsible for ischemic neuronal death and brain injury, and that suppression of AMPK is capable of exerting protective effects against ischemic stroke.

It is worth noting, however, that although inhibition of AMPK by Compound C could alleviate neuronal apoptosis and brain injury induced by ischemia/hypoxia, Compound C also inhibits several other kinases and is therefore non-specific to AMPK (Dasgupta & Seibel. 2018). In particular, it is reported that Compound C could inhibit the VEGF type 2 receptor and thus disrupt angiogenesis during zebrafish development, and it also blocked VEGF-induced tubular network formation in human umbilical vein endothelial cells (HUVEC) when incubated with HUVEC for 15 h (Hao et al. 2010). Therefore, inhibition of AMPK by Compound C might exert harmful effects on cerebral ischemia due to its influence on the angiogenesis post-stroke. In the present study, we utilized Compound C to treat Neuro-2a cells and mice for a short time in the early stage of OGD exposure or dMCAO, and found that Compound C treatment had a protective effect against ischemic damage, at least in part, through the reduction of AMPK-mediated Drp1 phosphorylation. These findings are consistent with previous observations showing that suppression of AMPK by Compound C reduced infarct size and improved neurological outcome (Ma et al. 2015; Nam et al. 2013). These different results could be attributable to the differences in the context of the timing, duration, and amount of Compound C used in the experiments. Certainly, given the Compound C inhibition on other kinases, further investigation will be necessary to develop a highly selective inhibitor for AMPK.

Additionally, some studies reported that AMPK is a protective molecule in ischemic stroke. For example, it has been shown that the activation of AMPK prior to injury enhanced the tolerance of neurons to ischemic/hypoxic insult(Huang et al. 2015; Jiang et al. 2018) and that inhibiting AMPK during OGD promoted neuronal survival, whereas inhibiting AMPK prior to OGD exacerbated cell death(Rousset et al. 2015). These differences are speculated to be caused by different ischemic degrees, durations, models and different cell types, or whether activating AMPK is harmful or beneficial is likely dependent on whether it is activated prior to or during the ischemic/hypoxic insult(Jiang et al. 2018). Therefore, more studies are required to elucidate the roles of AMPK in ischemic stroke in the future.

CDK5 is predominantly expressed in post-mitotic neurons(Wang et al. 2006) and plays an important role in the pathological process of neurological diseases, including Alzheimer’s disease (AD), Parkinson’s disease (PD), cerebral ischemic injury, amyotrophic lateral sclerosis, and Huntington’s disease(Ao et al. 2022; Chen et al. 2021). CDK5 has been known to be one of the upstream regulators for Drp1 phosphorylation(Chen et al. 2021) and its activation enhances mitochondrial fission via Drp1 phosphorylation at S616 in chronic ethanol exposure-induced cognitive impairment(Liu et al. 2022). Despite these advances, it remains unknown whether AMPK mediates the regulation of CDK5 on Drp1 phosphorylation. Here, we found that CDK5 expression and the phosphorylation of AMPK and Drp1 were significantly up-regulated in OGD-treated neuronal cells as well as in the ischemic brain tissues. Remarkably, the expression of Drp1 and GCN5L1 in the ischemic brain tissues of cdk5 knockout mice was down-regulated 3 days after dMCAO. Moreover, the interaction of GCN5L1 with Drp1 and thus Drp1 acetylation were decreased in the ischemic brain tissues of cdk5-deficient mice. These findings imply that CDK5 up-regulation and AMPK activation by hypoxia/ischemia are necessary for the phosphorylation of Drp1 in neuronal cells, i.e., that AMPK mediates the regulation of CDK5 on Drp1 phosphorylation. Altogether, our results disclose a novel function of GCN5L1 in regulating Drp1 acetylation and identify a previously unrecognized CDK5-AMPK-GCN5L1 pathway that mediates the acetylation of Drp1 in ischemic/hypoxic neuronal cells as well as in ischemic brain tissues.

## Electronic supplementary material

Below is the link to the electronic supplementary material.

Supplementary Material 1

Supplementary Material 2

## Data Availability

No datasets were generated or analysed during the current study.

## Abbreviations

dMCAO: The distal middle cerebral artery occlusion
TTC: 2,3,5-Triphenyltetrazolium chloride
RT-qPCR: Real-time quantitative polymerase chain reaction
MtROS: Mitochondrial ROS
