# Supplementary material for: Drp1 acetylation mediated by CDK5-AMPK-GCN5L1 axis promotes cerebral ischemic injury via facilitating mitochondrial fission
Source: Mol Med. 2024 Oct 10;30:173. doi: 10.1186/s10020-024-00948-y (PMC11468353; doi:10.1186/s10020-024-00948-y)
Supplement: Supplementary file 1 — Supplementary Material 1 [file 10020_2024_948_MOESM1_ESM.pdf]

Supplementary Fig. 1.

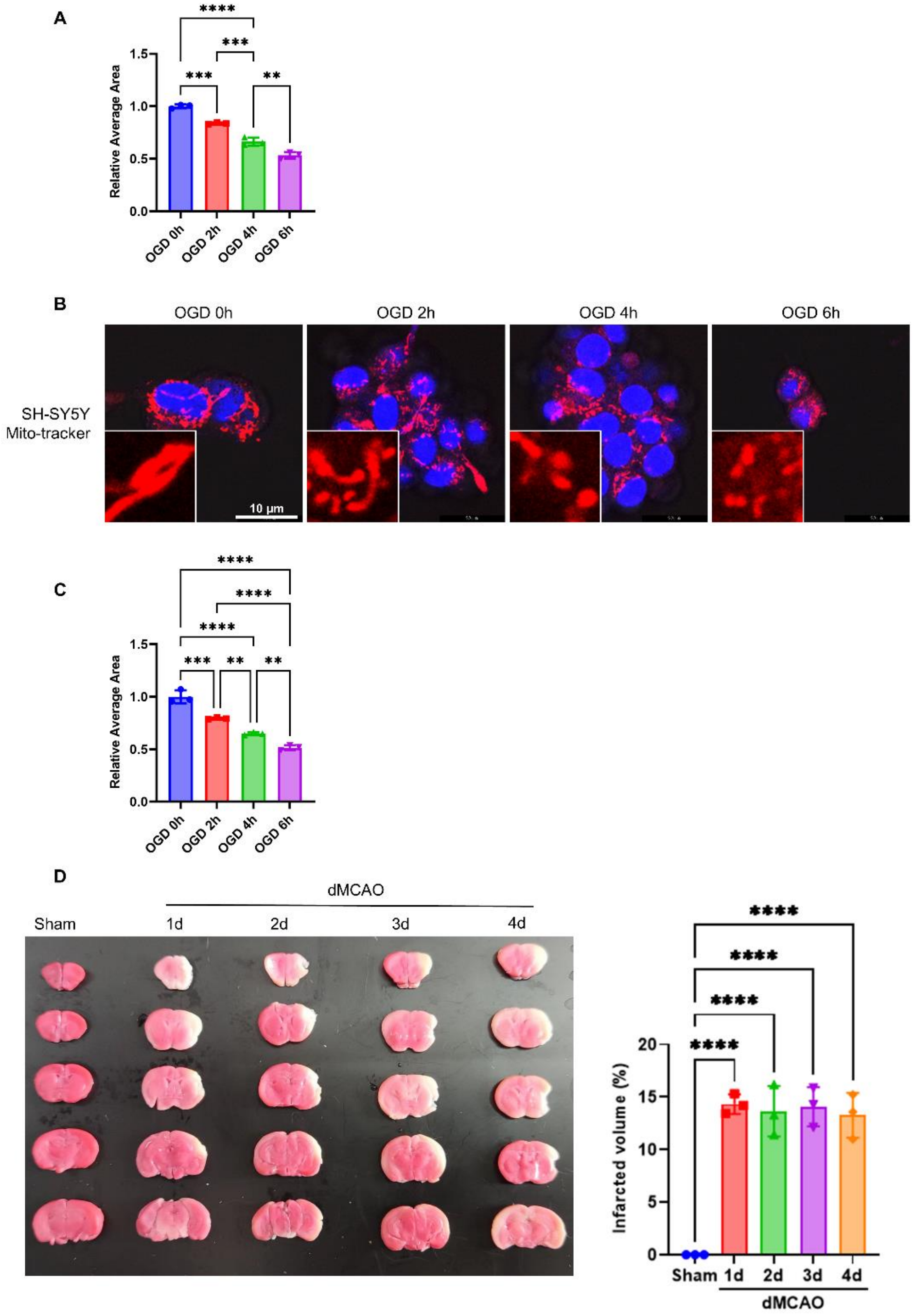

Supplementary Fig. 1. Ischemia/hypoxia induces mitochondrial fission, mtROS production and apoptosis in neuronal cells and brain tissues. (A) Mitochondrial morphology of Neuro-2a cells in Fig. 1A was quantified by Average Area (AA), by Image J software (n=3). (B) Mitochondrial morphology was visualized by 50 nM MitoTracker Red staining and observed by confocal microscopy in SH-SY5Y cells treated with OGD for different times. Scale bars represent 10 μm. (C) Mitochondrial

morphology of SH-YY5Y cells were quantified by Average Area (AA), by Image J software (n=3). (D) Representative TTC-stained brain sections and quantitative analysis of infarct volume. Data are represented as mean ± SD, $^{**}P<0.01$, $^{***}P<0.005$, $^{****}P<0.0001$, *P*-value was determined by one-way ANOVA with Dunnett's post hoc correction.

Supplementary Fig. 2.

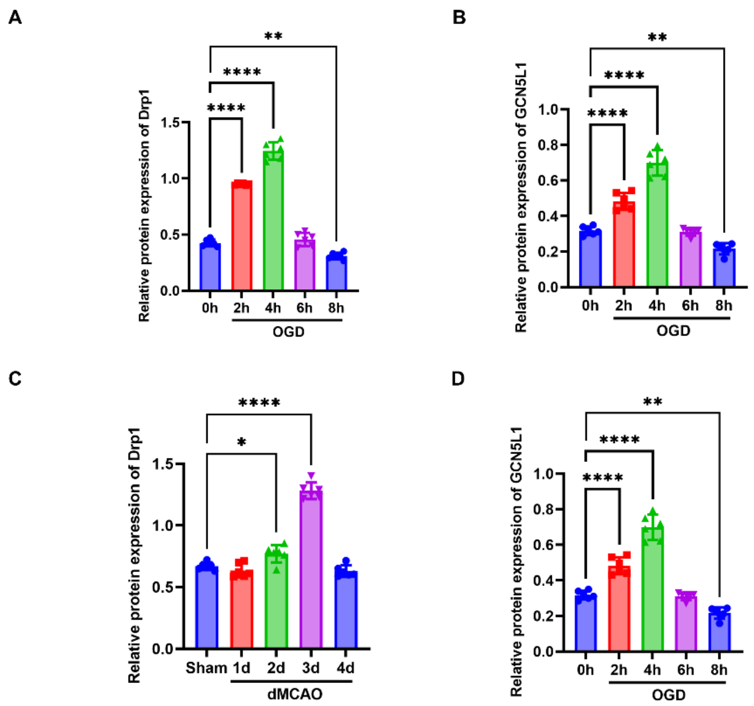

Supplementary Fig. 2. Drp1 and GCN5L1 are upregulated in OGD-treated neuronal cells and the ischemic brain tissues induced by dMCAO. (A) Quantitative analysis of the western blot of Drp1 in Fig. 2A in Neuro-2a cells treated with OGD for different times. (B) Quantitative analysis of the western blot of GCN5L1 in Fig. 2A in Neuro-2a cells treated with OGD for different times. (C) Quantitative analysis of the western blot of Drp1 in Fig. 2D in brain sections of dMCAO and control mice. (D) Quantitative analysis of the western blot of GCN5L1 in Fig. 2D in brain sections of dMCAO and control mice. Data are represented as mean ± SD, ***P*<0.01, *****P*<0.0001, *P*-value was determined by one-way ANOVA with Dunnett's post hoc correction.

Supplementary Fig. 3.

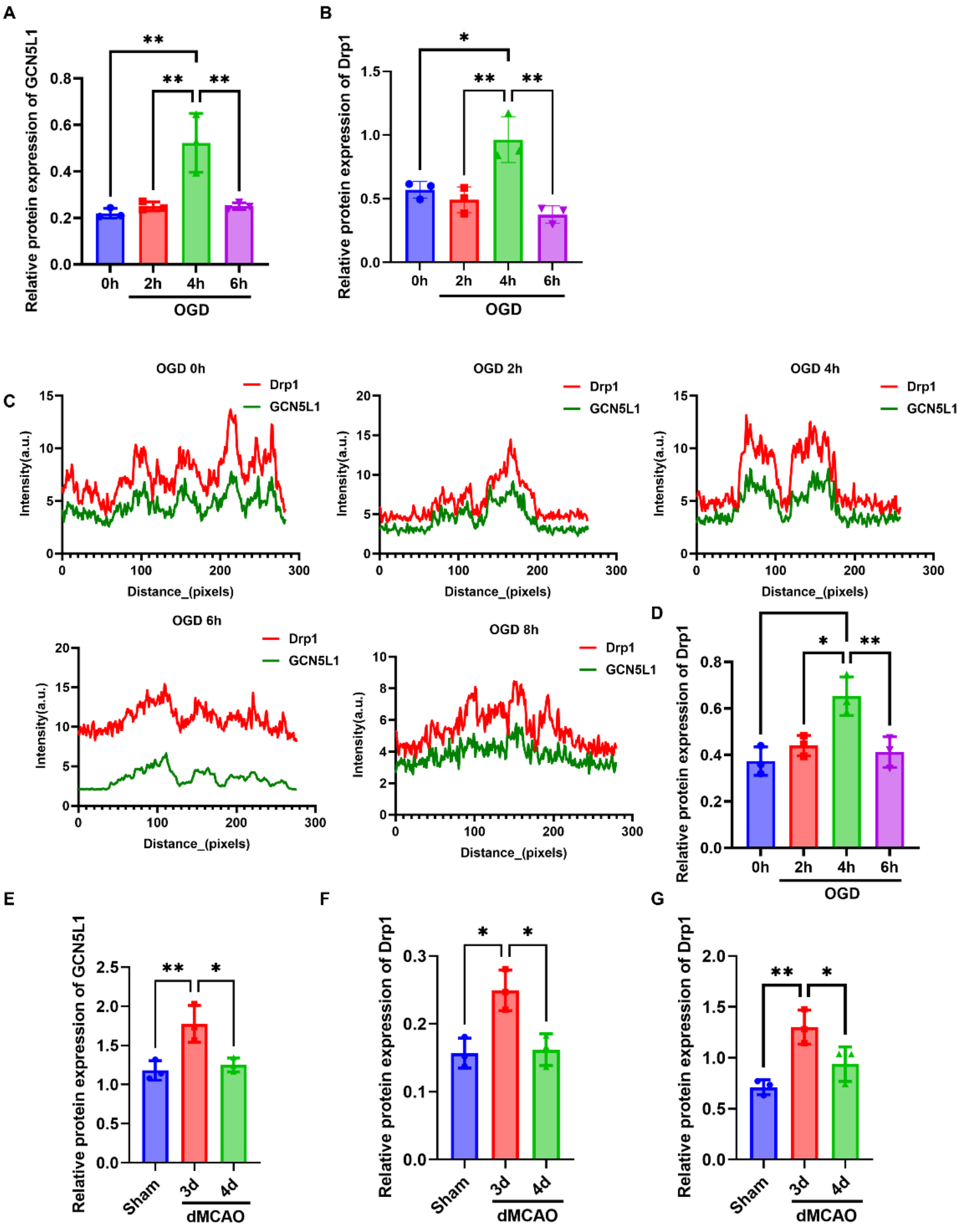

Supplementary Fig. 3. Ischemia/hypoxia facilitates Drp1 interaction with GCN5L1 and leads to Drp1 acetylation. (A) Quantitative analysis of the co-immunoprecipitation of GCN5L1 in Fig. 3A in neuro-2a cells treated with OGD for different times. (B) Quantitative analysis of the co-immunoprecipitation of Drp1 in Fig. 3B in neuro-2a cells treated with OGD for different times. (C) Quantitative analysis of the co-localization between Drp1 and GCN5L1 in Fig. 3C in neuro-2a cells treated with OGD for different times. (D) Quantitative analysis of Drp1 using immunoprecipitates with acetyl-lys in Fig. 3D in neuro-2a cells treated with OGD for

different times. (E) Quantitative analysis of the co-immunoprecipitation of GCN5L1 in Fig. 3E in brain tissues of dMCAO and control mice. (F) Quantitative analysis of the co-immunoprecipitation of Drp1 in Fig. 3F in brain tissues of dMCAO and control mice. (G) Quantitative analysis of Drp1 using immunoprecipitates with acetyl-lys in Fig. 3G in brain tissues of dMCAO and control mice. Data are represented as mean ± SD, *$P < 0.05$, **$P < 0.01$, P-value was determined by one-way ANOVA with Dunnett's post hoc correction.

Supplementary Fig. 4.

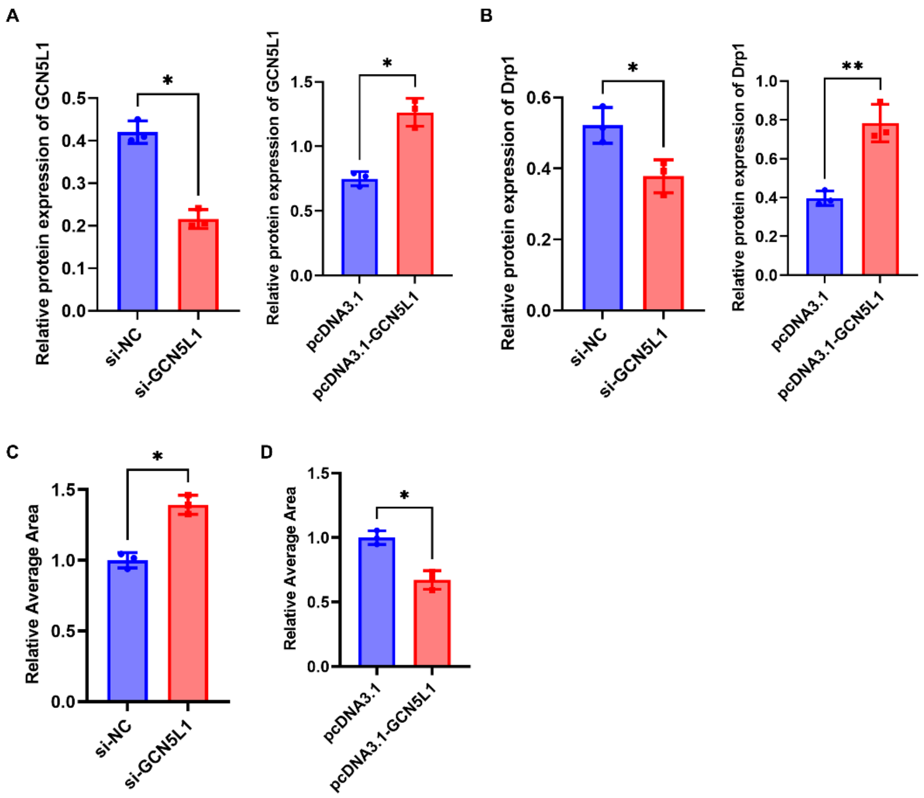

Supplementary Fig. 4. Knockdown or overexpression of GCN5L1 attenuates or enhances mitochondrial fission in neuronal cells. (A) Quantitative analysis of the western blot of GCN5L1 in Fig. 4B in si-GCN5L1 or pcDNA3.1-GCN5L1-transfected Neuro-2a cells. (B) Quantitative analysis of Drp1 using immunoprecipitates with acetyl-lys in Fig. 4C and 4D in si-GCN5L1 or pcDNA3.1-GCN5L1-transfected Neuro-2a cells. (C-D) Mitochondrial morphology of Neuro-2a cells with si-GCN5L1 or pcDNA3.1-GCN5L1-transfected in Fig. 4E and 4F was quantified by Average Area (AA), by Image J software (n=3). Data are represented as mean ± SD, *P <0.05, **P<0.01, P-value was determined by student t-test.

Supplementary Fig. 5.

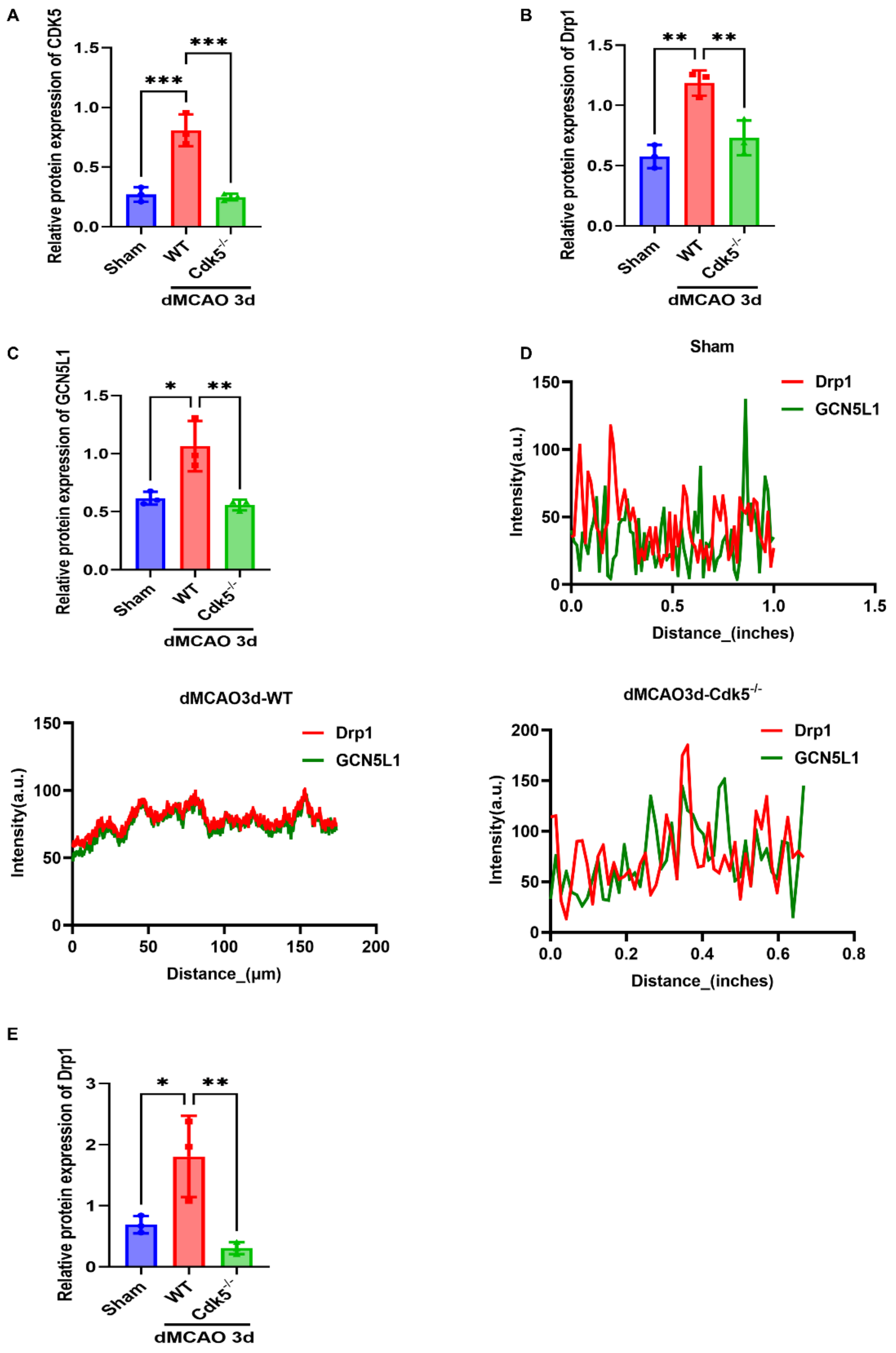

Supplementary Fig. 5. cdk5 deficiency protects brain tissues against ischemic injury in mice. (A-C) Quantitative analysis of the western blot of CDK5, Drp1 and GCN5L1 in Fig. 5C in ischemic brain tissues of WT and cdk5 knockout mice. (D) Quantitative analysis of the co-localization between Drp1 and GCN5L1 in Fig. 5E in ischemic brain tissues of WT and cdk5 knockout mice. (E) Quantitative analysis of Drp1 using

immunoprecipitates with acetyl-lys in Fig. 5F in ischemic brain tissues of WT and cdk5 knockout mice. (n=3) Data are represented as mean ± SD, **P* <0.05, ***P* <0.01, ****P*<0.005, *P*-value was determined by one-way ANOVA with Dunnett's post hoc correction.

Supplementary Fig. 6.

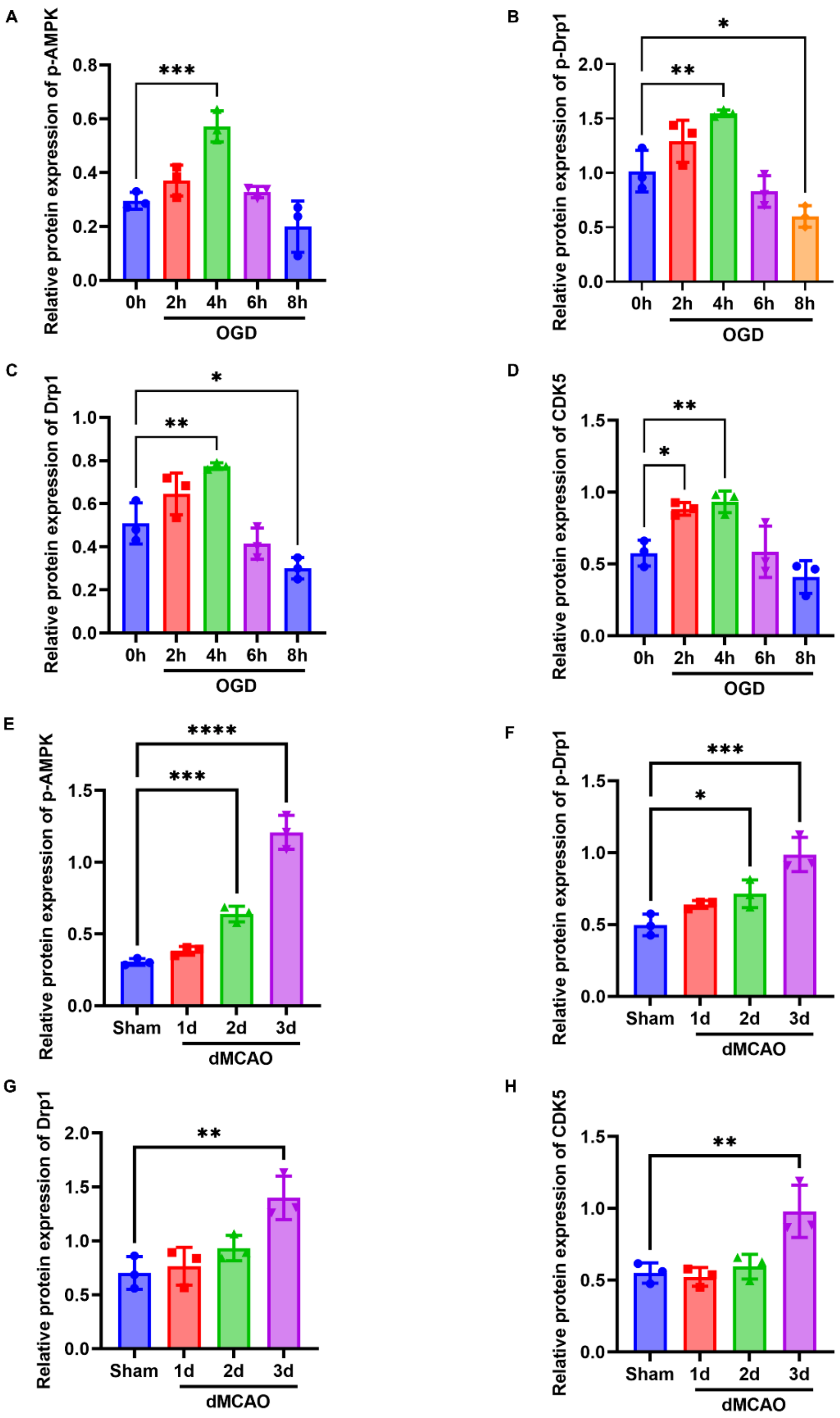

Supplementary Fig. 6. Ischemia/hypoxia upregulates CDK5 expression and induces the phosphorylation of AMPK and Drp1. (A-D) Quantitative analysis of the western blot of p-AMPK, p-Drp1, Drp1 and CDK5 in Fig. 6A in Neuro-2a cells treated with OGD for different times. (E-H) Quantitative analysis of the western blot of p-AMPK,

p-Drp1, Drp1 and CDK5 in Fig. 6C in brain sections of dMCAO and control mice. Data are represented as mean ± SD, **P*<0.05, ***P*<0.01, ****P*<0.005, *****P*<0.0001, *P*-value was determined by one-way ANOVA with Dunnett's post hoc correction.

Supplementary Fig. 7.

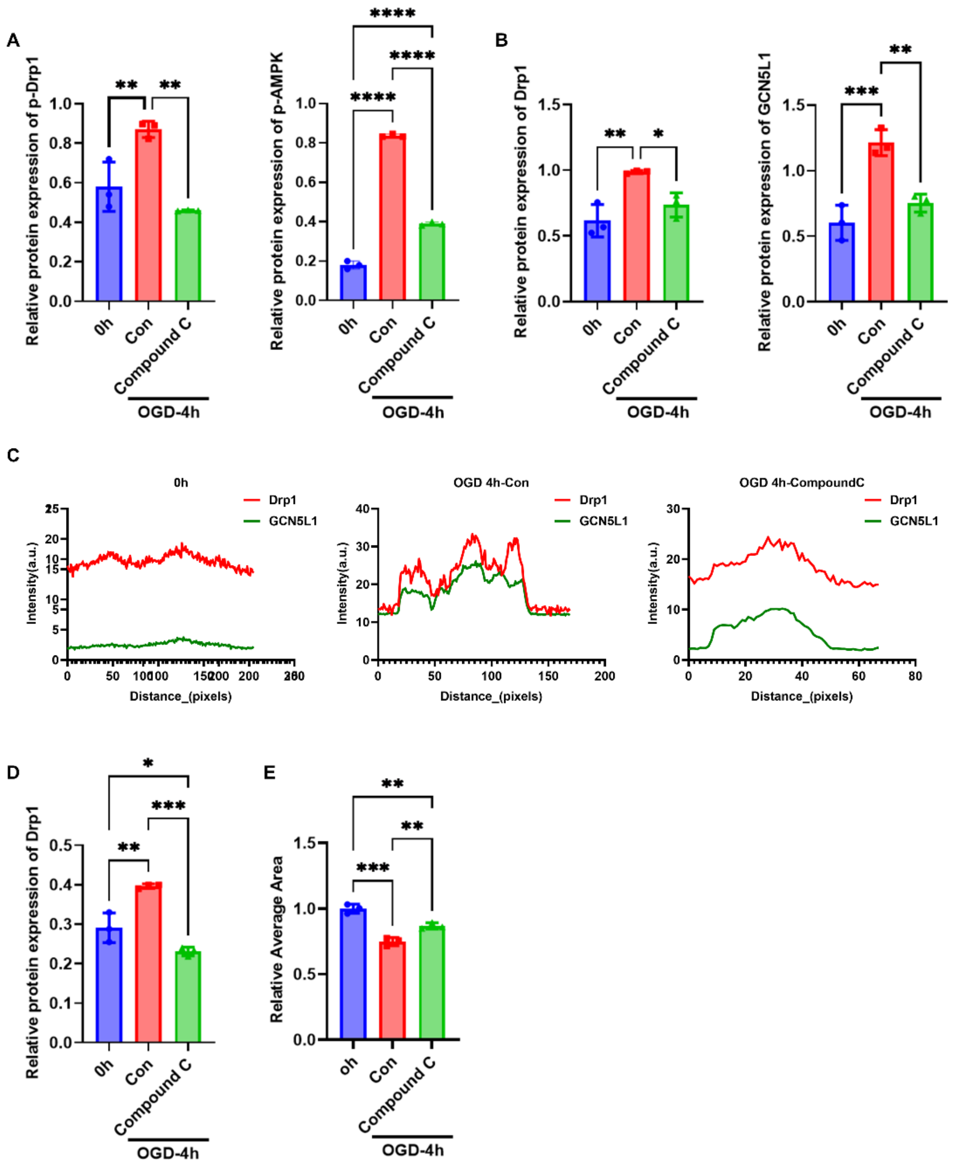

Supplementary Fig. 7. Inhibition of AMPK attenuates Drp1 acetylation and mitochondrial fission in OGD-treated neuronal cells. (A) Quantitative analysis of the western blot of p-Drp1 and p-AMPK in Fig. 7A in Neuro-2a cells treated with OGD and Compound C. (B) Quantitative analysis of the co-immunoprecipitation of Drp1 and GCN5L1 in Fig. 7C in neuro-2a cells treated with OGD and Compound C. (C) Quantitative analysis of the co-localization between Drp1 and GCN5L1 in Fig. 7D in neuro-2a cells treated with OGD and Compound C. (D) Quantitative analysis of Drp1 using immunoprecipitates with acetyl-lys in Fig. 7E in neuro-2a cells treated with OGD and Compound C. (E) Mitochondrial morphology of Neuro-2a cells treated with OGD and Compound C was quantified by Average Area (AA) in Fig. 7F, by Image J

software (n=3). Data are represented as mean ± SD, *P<0.05, **P <0.01, ***P<0.005, ****P<0.0001, P-value was determined by one-way ANOVA with Dunnett's post hoc correction.

Supplementary Fig. 8.

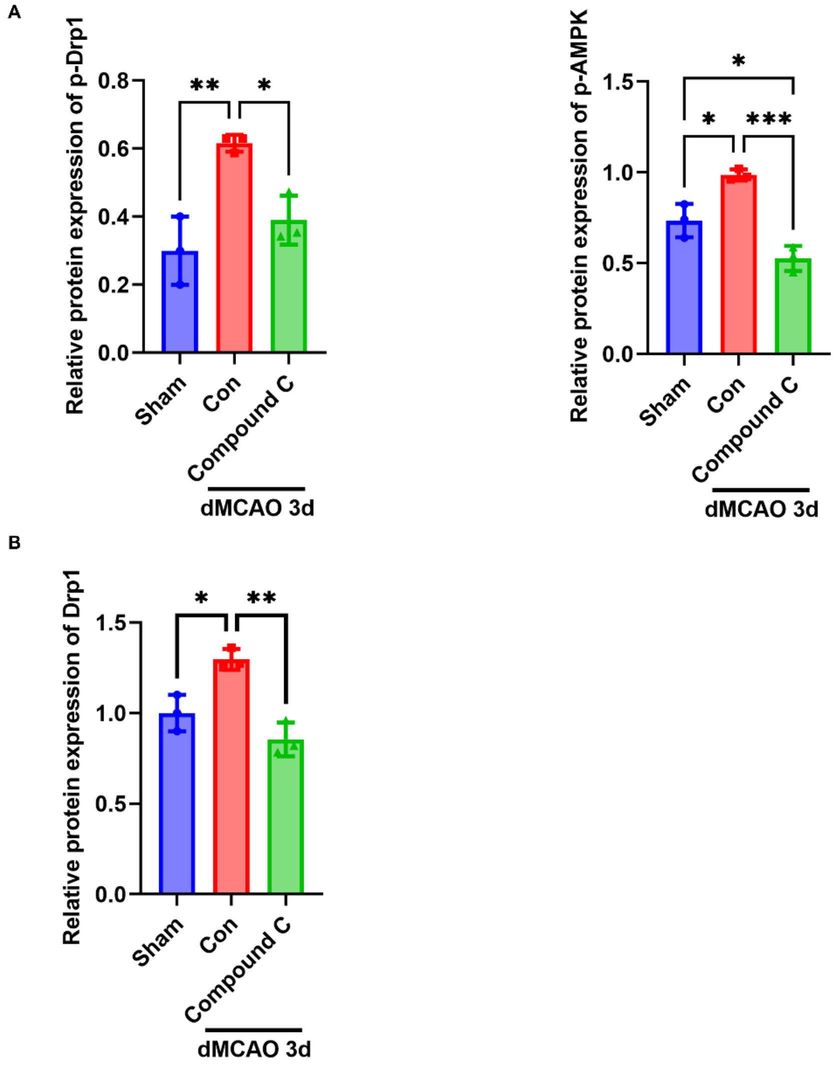

Supplementary Fig. 8. Inhibition of AMPK by Compound C protects brain tissues from ischemic damage in mouse models of dMCAO. (A) Quantitative analysis of the western blot of p-Drp1 and p-AMPK in Fig. 8B in brain tissues of dMCAO and control mice treated with or without Compound C. (B) Quantitative analysis of Drp1 using immunoprecipitates with acetyl-lys in Fig. 8D in brain tissues of dMCAO and control mice treated with or without Compound C. Data are represented as mean ± SD, **P*<0.05, ***P*<0.01, ****P*<0.005, *P*-value was determined by one-way ANOVA with Dunnett’s post hoc correction
