# Supplementary material for: Drp1 acetylation mediated by CDK5-AMPK-GCN5L1 axis promotes cerebral ischemic injury via facilitating mitochondrial fission
Source: Mol Med. 2024 Oct 10;30:173. doi: 10.1186/s10020-024-00948-y (PMC11468353; doi:10.1186/s10020-024-00948-y)
Supplement: Supplementary file 2 — Supplementary Material 2 [file 10020_2024_948_MOESM2_ESM.pdf]

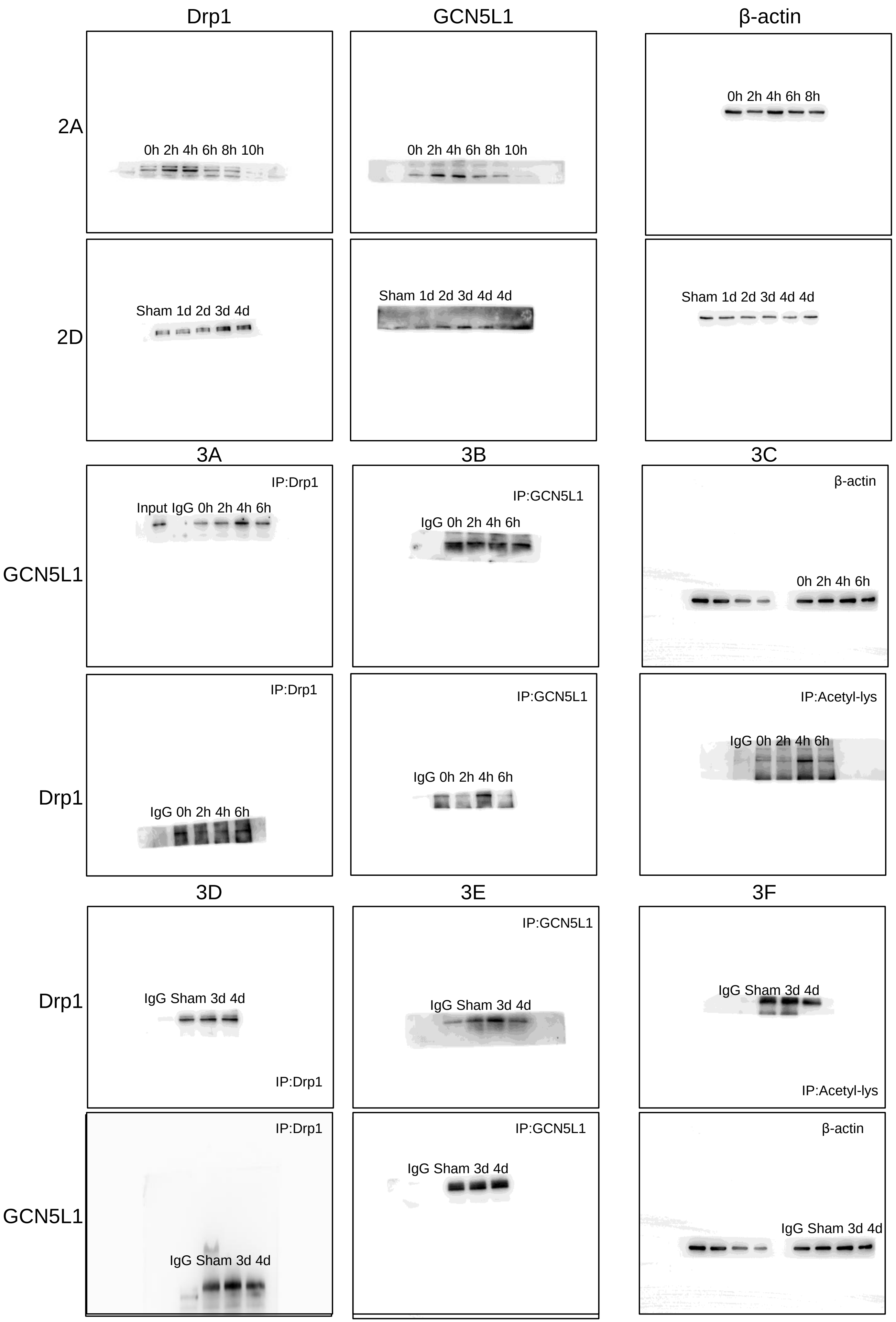
Drp1
GCN5L1
β-actin
2A
0h 2h 4h 6h 8h 10h
0h 2h 4h 6h 8h 10h
0h 2h 4h 6h 8h
2D
Sham 1d 2d 3d 4d
Sham 1d 2d 3d 4d 4d
Sham 1d 2d 3d 4d 4d
3A
3B
3C
GCN5L1
IP:Drp1
Input IgG 0h 2h 4h 6h
IP:GCN5L1
IgG 0h 2h 4h 6h
β-actin
0h 2h 4h 6h
Drp1
IP:Drp1
IgG 0h 2h 4h 6h
IP:GCN5L1
IgG 0h 2h 4h 6h
IP:Acetyl-lys
IgG 0h 2h 4h 6h
3D
3E
3F
Drp1
IgG Sham 3d 4d
IP:Drp1
IP:GCN5L1
IgG Sham 3d 4d
IgG Sham 3d 4d
IP:Acetyl-lys
GCN5L1
IP:Drp1
IgG Sham 3d 4d
IP:GCN5L1
IgG Sham 3d 4d
β-actin
IgG Sham 3d 4d

4B

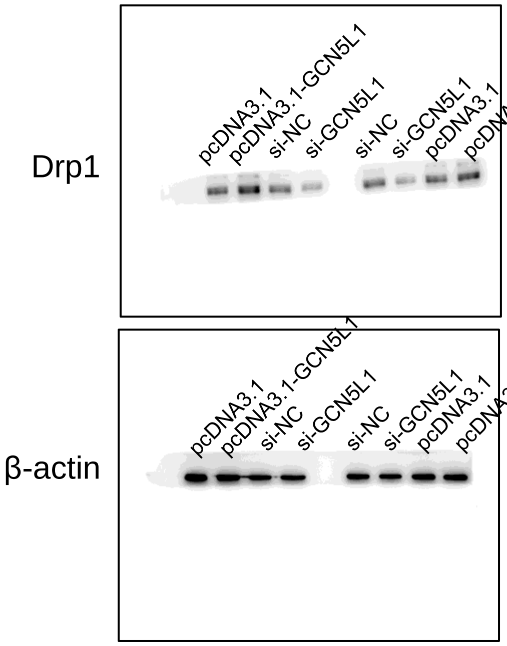
Drp1
pcDNA3.1
pcDNA3.1-GCN5L1
si-NC
si-GCN5L1
si-NC
si-GCN5L1
pcDNA3.1
pcDNA3.1-GCN5L1
β-actin

4C

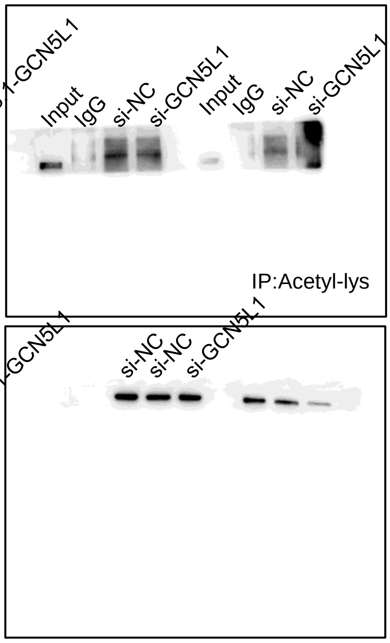
Input
IgG
si-NC
si-GCN5L1
Input
IgG
si-NC
si-GCN5L1
IP:Acetyl-lys
si-NC
si-NC
si-GCN5L1

4D

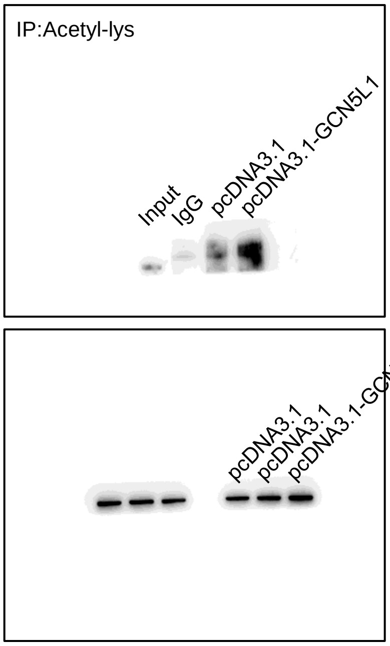
IP:Acetyl-lys
Input
IgG
pcDNA3.1
pcDNA3.1-GCN5L1
pcDNA3.1
pcDNA3.1
pcDNA3.1-GCN5L1

5C

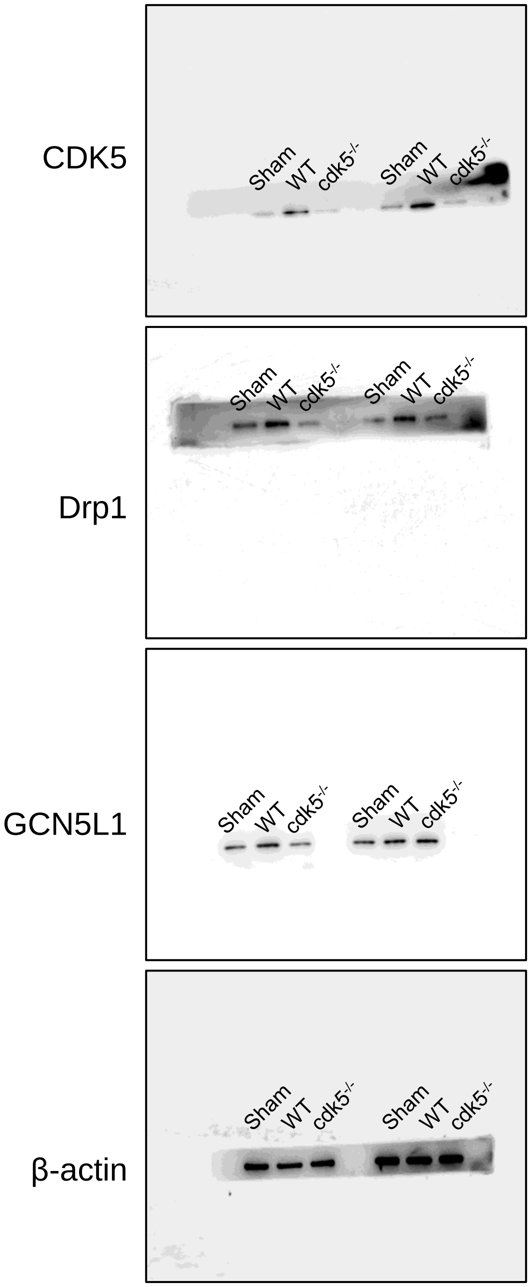
CDK5
Sham
WT
cdk5-/-
Drp1
GCN5L1
β-actin

5F

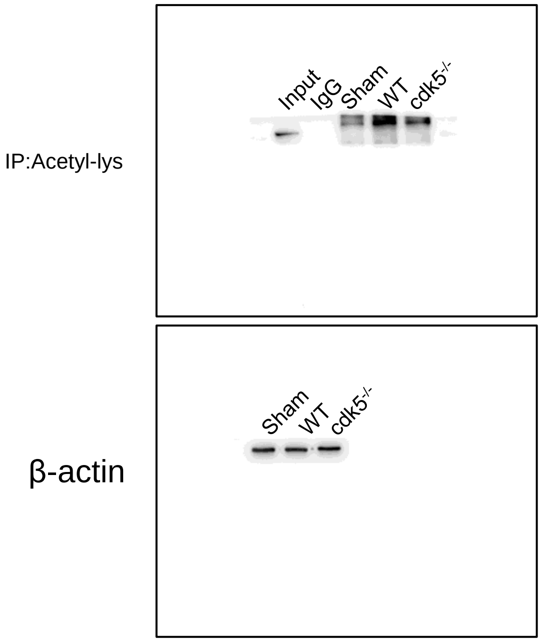
IP:Acetyl-lys
Input
IgG
Sham
WT
cdk5-/-
β-actin

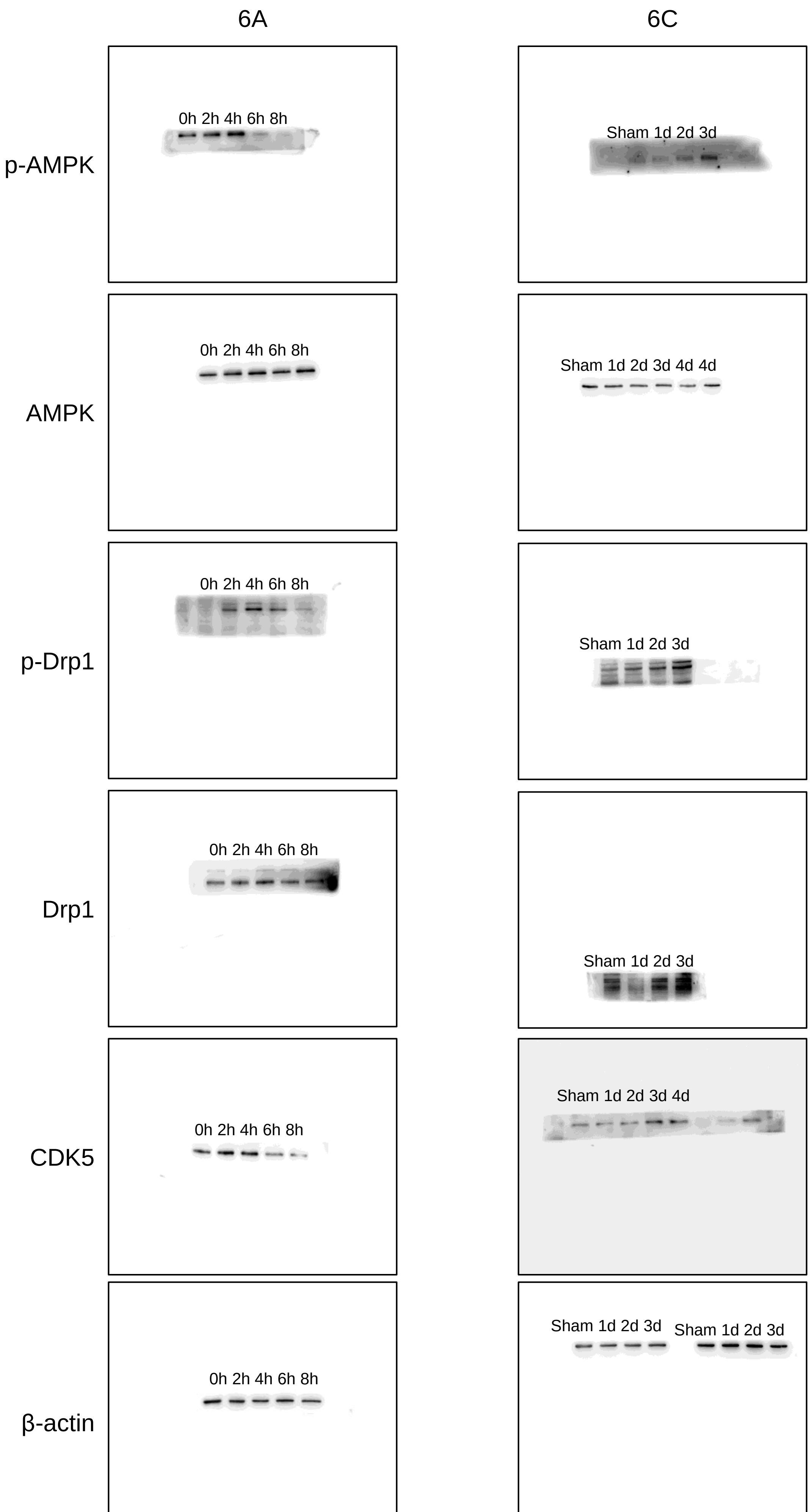
6A
6C
p-AMPK
0h 2h 4h 6h 8h
Sham 1d 2d 3d
AMPK
0h 2h 4h 6h 8h
Sham 1d 2d 3d 4d 4d
p-Drp1
0h 2h 4h 6h 8h
Sham 1d 2d 3d
Drp1
0h 2h 4h 6h 8h
Sham 1d 2d 3d
CDK5
0h 2h 4h 6h 8h
Sham 1d 2d 3d 4d
β-actin
0h 2h 4h 6h 8h
Sham 1d 2d 3d
Sham 1d 2d 3d

7A

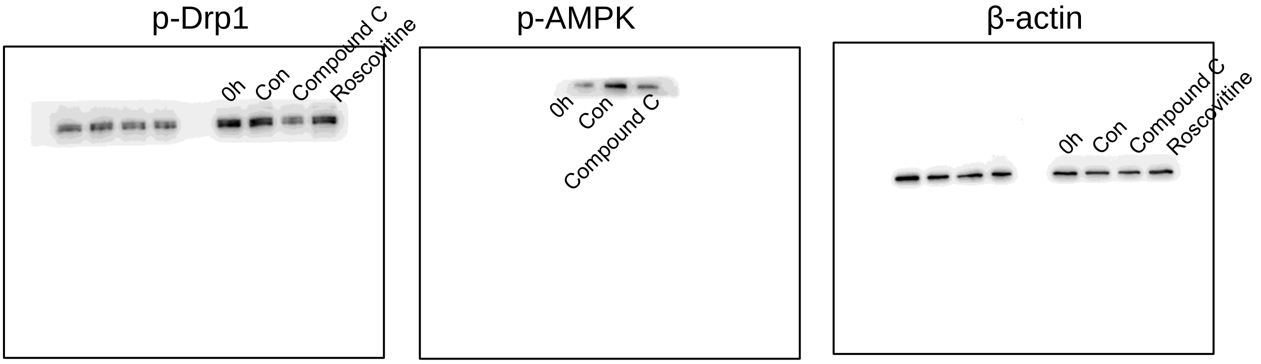
p-Drp1
0h
Con
Compound C
Roscovitine
p-AMPK
0h
Con
Compound C
β-actin
0h
Con
Compound C
Roscovitine

7C

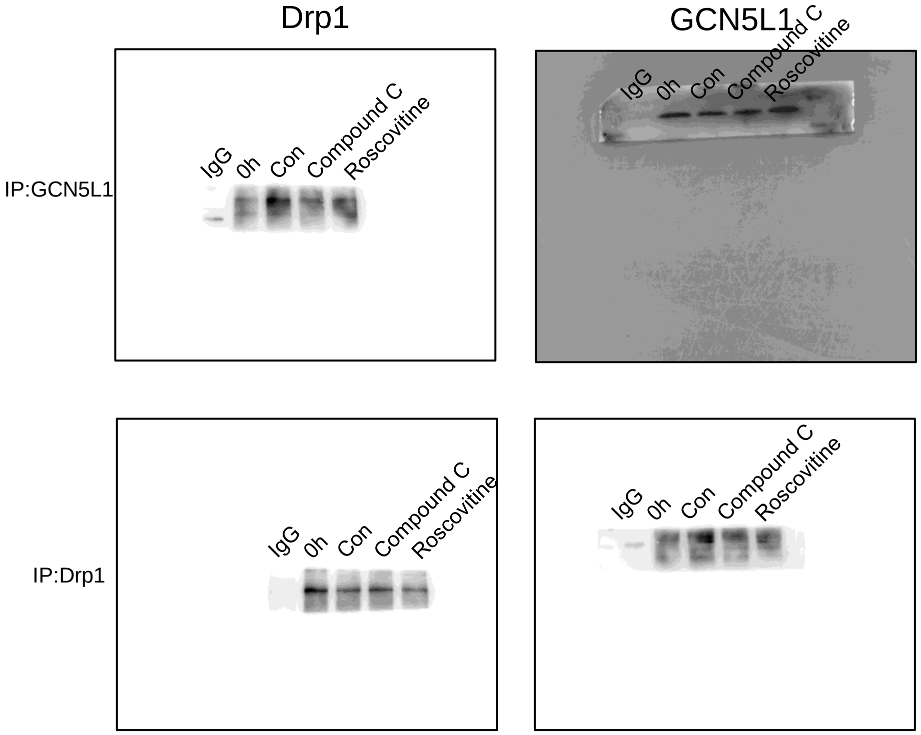
Drp1
GCN5L1
IP:GCN5L1
IgG
0h
Con
Compound C
Roscovitine
IgG
0h
Con
Compound C
Roscovitine
IP:Drp1
IgG
0h
Con
Compound C
Roscovitine
IgG
0h
Con
Compound C
Roscovitine

7E

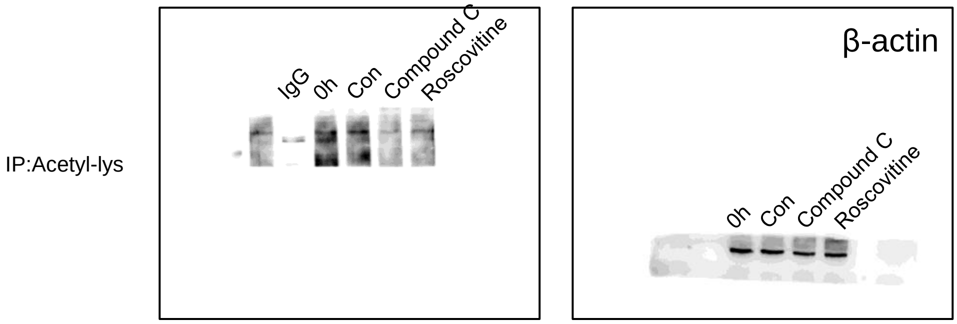
IP:Acetyl-lys
IgG
0h
Con
Compound C
Roscovitine
β-actin
0h
Con
Compound C
Roscovitine

8B

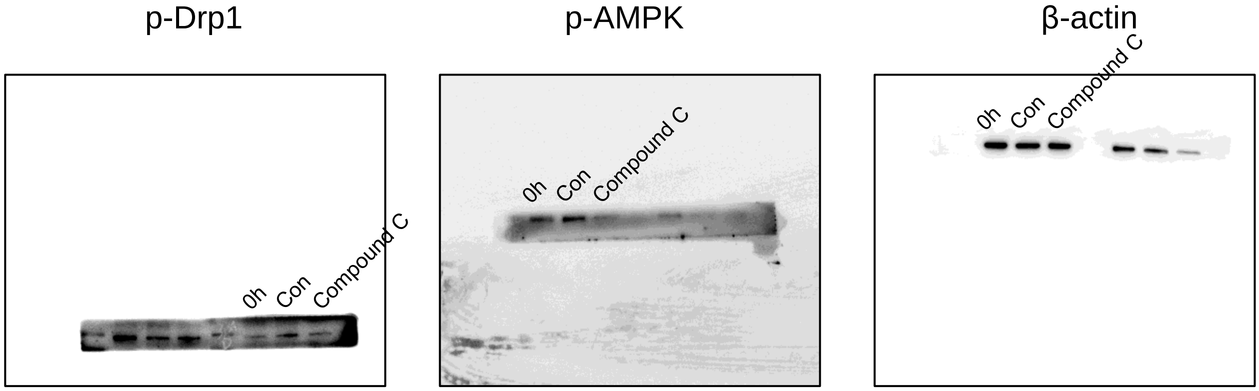
p-Drp1
0h
Con
Compound C
p-AMPK
0h
Con
Compound C
β-actin
0h
Con
Compound C

8D

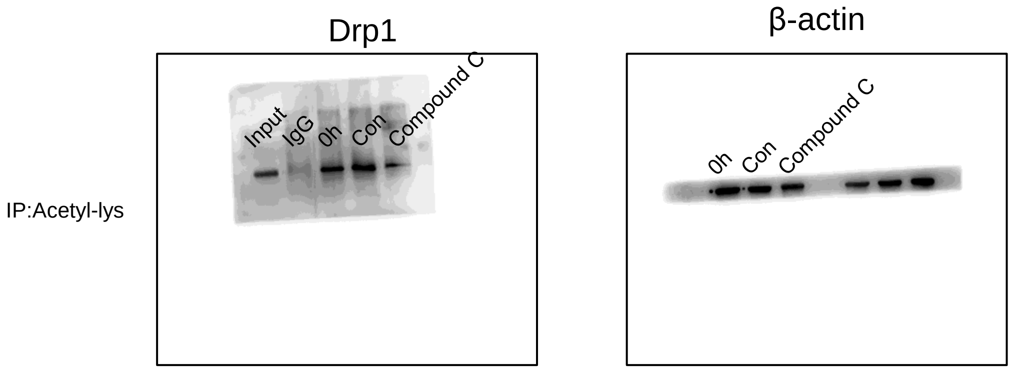
IP:Acetyl-lys
Drp1
Input
IgG
0h
Con
Compound C
β-actin
0h
Con
Compound C
